# Dual-Task Costs in Working Memory: An Adversarial Collaboration

**DOI:** 10.1037/xlm0000668

**Published:** 2018-11-08

**Authors:** Jason M. Doherty, Clement Belletier, Stephen Rhodes, Agnieszka Jaroslawska, Pierre Barrouillet, Valerie Camos, Nelson Cowan, Moshe Naveh-Benjamin, Robert H. Logie

**Affiliations:** 1 Department of Psychology, The University of Edinburgh; 2 Department of Psychology, University of Fribourg; 3 Department of Psychology, University of Missouri; 4 Department of Psychology, The University of Edinburgh; 5 Department of Psychology, University of Geneva; 6 Department of Psychology, University of Fribourg; 7 Department of Psychology, University of Missouri; 8 Department of Psychology, The University of Edinburgh

**Keywords:** working memory, multiple-component, TBRS, embedded processes, adversarial collaboration

## Abstract

Theories of working memory often disagree on the relationships between processing and storage, particularly on how heavily they rely on an attention-based limited resource. Some posit separation and specialization of resources resulting in minimal interference to memory when completing an ongoing processing task, while others argue for a greater overlap in the resources involved in concurrent tasks. Here, we present four experiments that investigated the presence or absence of dual-task costs for memory and processing. The experiments were carried in an adversarial collaboration in which researchers from three opposing theories collaboratively designed a set of experiments and provided differential predictions in line with each of their models. Participants performed delayed recall of aurally and visually presented letters and an arithmetic verification task either as single tasks or with the arithmetic verification task between presentation and recall of letter sequences. Single- and dual-task conditions were completed with and without concurrent articulatory suppression. A consistent pattern of dual-task and suppression costs was observed for memory, with smaller or null effects on processing. The observed data did not fit perfectly with any one framework, with each model having partial success in predicting data patterns. Implications for each of the models are discussed, with an aim for future research to investigate whether some combination of the models and their assumptions can provide a more comprehensive interpretation of the pattern of effects observed here and in relevant previous studies associated with each theoretical framework.

The term *working memory* refers to the process or collection of processes responsible for the complex cognitive coordination necessary for everyday human thoughts and actions. Researchers generally agree about the importance of working memory for human cognition. There is also general agreement that it supports the ready availability of a small amount of information in support of current tasks, and has a key role in updating and processing that information moment to moment (e.g., Cowan, 2017; Logie & Cowan, 2015). However, there are multiple different definitions of working memory (see Cowan, 2017, for a discussion), and each definition gives rise to different theoretical assumptions and different experimental paradigms designed to test those assumptions. Contrasting results across labs might then reflect the specific experimental paradigms adopted, and theoretical debates may be based on differences that are more apparent than real (Logie, 2011). Rarely do researchers who assume different definitions of working memory adopt the exact same paradigm to directly test their contrasting predictions.

We present four experiments that addressed the debate about what limits the capacity of working memory to undertake both memory maintenance and ongoing processing. Unlike most studies in this area, the experiments were carried out across different labs within an “adversarial collaboration” in which the coauthors agreed on a common experimental paradigm to test predictions from their contrasting, and well-established theoretical frameworks for working memory. The experiments described here are part of a larger project called Working Memory Across the Adult Life Span: An Adversarial Collaboration (WoMAAC; https://womaac.psy.ed.ac.uk). Specifically, these frameworks are referred to as the multiple-component model (MCM; Baddeley & Logie, 1999; Logie, 2011, 2016), time-based resource sharing (TBRS; Barrouillet & Camos, 2010, 2015), and embedded processes (EP; Cowan, 1999, 2005). This approach allows a more direct test of the different predictions than is possible across different studies, with the aim of contributing new insights, both theoretically and empirically, to this important area of cognition. First, we give an overview of each of the three theoretical frameworks that motivated our experiments, and then go on to describe the expectations from each for the series of experiments that follow. All of the predictions from each theory, and the experimental methods, were preregistered on the Open Science Framework (OSF, see the project page at https://bit.ly/2KTKMgb).

## Multiple-Component Model (MCM)

The MCM assumes a coordinated system of specialized cognitive resources serving specific functions in online cognition. The model specifies separate components for storage and processing, with distinct stores based on modality-specific codes that need not match the modality of presentation. For example, words may be stored as visual codes or as phonological or semantic codes, regardless of whether they are presented visually or aurally, and nonverbal stimuli such as shapes and colors may be stored as visual codes or as phonological or semantic codes for the associated names. Originally (Baddeley, 1986; Baddeley & Hitch, 1974), a central executive was proposed as a domain-general processing and control mechanism, but subsequently (Baddeley, 1996; Logie, 2016), a number of separate executive functions were proposed, such as inhibition, updating, task-switching (Miyake et al., 2000), dual-tasking (Logie, Cocchini, Della Sala, & Baddeley, 2004; MacPherson, Della Sala, Logie, & Wilcock, 2007), and the manipulation of mental images (Borst, Niven, & Logie, 2012; Van Der Meulen, Logie, & Della Sala, 2009). Executive functions have therefore been suggested to be emergent properties of the interaction between these multiple functions (Logie, 2011, 2016).

The phonological loop has been proposed as a temporary store for serial ordered phonological codes (e.g., Baddeley, 1992). Items stored within the phonological loop are said to be vulnerable to interference among themselves due to phonological similarity (Conrad & Hull, 1964) and interference from asking participants to repeat aloud an irrelevant word (e.g., the–the–the) while encoding or retaining verbal sequences (a technique known as articulatory suppression [AS]), as well as from presentation of irrelevant speech (Salamé & Baddeley, 1982). While the limited capacity store can maintain small list lengths without any attentional cost, the MCM also proposes a separate subvocal rehearsal mechanism that can “boost” performance. Maintenance of longer lists through subvocal rehearsal has been found to be affected by a number of temporal factors, such as the length of words in a sequence and individual reading and speech rates (Baddeley, Thomson, & Buchanan, 1975; Hulme, Thomson, Muir, & Lawrence, 1984), although some recent studies have debated this issue (Guitard & Tolan, 2018; Jalbert, Neath, Bireta, & Surprenant, 2011; Oberauer, Farrell, Jarrold, & Lewandowsky, 2016). The links between memory performance and phonological characteristics of the to-be-remembered items are therefore argued as evidence for a specific verbal store. Additional evidence has come from studies of brain damaged individuals who appear to have very specific impairments of short-term retention of phonological sequences (Shallice & Warrington, 1970; Vallar & Baddeley, 1984).

The visual cache is said to store an array of visual items or a single visual item that may vary in complexity (Logie, 1995, 2003, 2011). The broader concept of visuospatial working memory is assumed to comprise separable resources and mechanisms dedicated to visual and spatial information (Logie, 2011; Logie & Marchetti, 1991; Logie & Pearson, 1997). Evidence for separate visual and spatial components also comes from the finding that spatial and visual memory spans increase at different rates with age during childhood, and are poorly correlated within age groups (Logie & Pearson, 1997).

While separate stores for verbal and visuospatial material are assumed by the MCM, the theory also states that material is often recoded for storage in other formats. For example, evidence that verbal material is represented in memory in the form of the visual appearance of the letters comes from the presence of visual similarity effects in serial written recall for visually presented verbal materials (Logie, Della Sala, Wynn, & Baddeley, 2000; Logie, Saito, Morita, Varma, & Norris, 2016; Saito, Logie, Morita, & Law, 2008), and other evidence has pointed to the use of verbal labels for abstract visual patterns (Brown & Wesley, 2013). MCM also assumes that different participants may approach tasks in multiple different ways that may not include phonological or visuospatial rehearsal mechanisms, using strategies such as employing mnemonics for remembering lists of words (Logie, Della Sala, Laiacona, Chalmers, & Wynn, 1996). In sum, working memory is viewed as a set of mental tools that can be applied in different combinations to support task performance, and the same task may be performed in different ways depending on which combination of working memory components are deployed.

The structure of working memory proposed by the MCM assumes a separation of processing and storage functions. In their seminal article, Baddeley and Hitch (1974) investigated the effect of concurrent memory load on processing tasks (e.g., sentence verification/comprehension, logical statement verification), and found that dual-task costs to processing were only observed at longer list lengths, and that greater interference effects than those observed should be expected if both storage and processing relied on a single limited resource. This argument has been made in a number of subsequent studies citing small or null effects as evidence for separate resources for each type of task (e.g., Doherty & Logie, 2016; Duff & Logie, 1999, 2001). Evidence for the separation of memory and processing is further provided by reports of low correlations between measures of memory span and measures of processing span (e.g., Daneman & Hannon, 2007; Logie & Duff, 2007; Waters & Caplan, 1996). Neuropsychological studies have also been used to argue for a dual-tasking ability based on coordination of multiple components; for example, Logie et al. (2004) and MacPherson et al. (2007) identified a specific dual-task deficit in Alzheimer’s patients that was not present in younger and older healthy controls. A key feature of dual-tasking studies within the MCM framework is that the cognitive demand of each task is adjusted (titrated) to the ability each individual participant, and this measured single-task ability is used to set the demand level both when performing each task on its own and when performing the two tasks together. This is done to ensure that any dual-task effect can be attributed specifically to the dual-task condition, and not because the individual-tasks were simply set at too high a level for the participant (for a more detailed discussion, see Logie et al., 2004).

## Time-Based Resource Sharing (TBRS) Model

The TBRS model assumes that both functions of working memory, processing and storage, rely in part on a shared, general-purpose, limited capacity attentional resource. Because a central bottleneck constrains cognitive operations to take place one at a time, when attention is occupied by processing it is no longer available for maintaining memory traces and so these traces suffer from temporal decay and interference. However, decayed memory traces may be restored through attentional refreshing when attention is available during pauses in processing. While temporary verbal memory can be bolstered by subvocal rehearsal in a phonological loop, performance is highly dependent on access to the focus of attention. The empirical basis for the theory is a number of observations of how the demand of a secondary processing task is inversely correlated with memory performance in a dual-task complex span paradigm (see Barrouillet & Camos, 2015, for a review). This attentional demand of a processing task is discussed in terms of its “cognitive load,” which refers to the proportion of time the processing task captures attention and therefore diverts the focus away from maintenance of temporary memory traces. Crucially, the TBRS model differentiates itself from pure decay-based theories of short-term forgetting in stating that it is not the overall duration of the processing component that matters but rather how much time between processing items is available for maintaining the representations of the memoranda.

TBRS research has demonstrated how cognitive load can be increased by increasing the number of retrievals from long-term memory (LTM; or the number of responses required by a secondary task), increasing the time taken to respond to each item of a distractor task, and decreasing the time of the processing period while keeping other factors constant (resulting in a smaller proportion of the time being available to refresh memory traces). These manipulations all result in higher cognitive load and thus poorer memory performance (e.g., Barrouillet, Bernardin, & Camos, 2004; Barrouillet, Bernardin, Portrat, Vergauwe, & Camos, 2007).

Attentional refreshing, the specific process that is interrupted by high cognitive load tasks, is described as separate from the subvocal rehearsal that is assumed to take place in the phonological loop (for reviews, see Camos, Lagner, & Barrouillet, 2009; Camos, Lagner, & Loaiza, 2017). Supporting evidence from brain imaging studies shows different activation patterns for each form of maintenance (Raye, Johnson, Mitchell, Greene, & Johnson, 2007; Trost & Gruber, 2012). The TBRS model states that refreshing can be actively or passively engaged depending on whether subvocal rehearsal is available or effective given task parameters or indeed whether participants are instructed to rehearse or refresh (Camos, Mora, & Oberauer, 2011). In the same way as processing prevents refreshing, refreshing activities postpone processing, as Vergauwe, Camos, and Barrouillet (2014) observed a slowing of processing task responses with increasing memory loads (see also Chen & Cowan, 2009). It is important to note that this effect occurs only when the phonological loop is unavailable (e.g., under AS) or when its capacity is exceeded. Importantly, the same study by Vergauwe et al. (2014) provided evidence that, contrary to verbal information for which a domain-specific storage system exists (i.e., the phonological loop), visuospatial information is not maintained by any domain-specific storage system and so its maintenance relies entirely on attention (Morey & Bieler, 2013; see also Morey, Morey, van der Reijden, & Holweg, 2013).

## Embedded Processes (EP)

The EP model, in its iterations over the years, has been developed to account for a wide range of empirical findings within a single framework (Cowan, 1988, 1999, 2005, 2008, 2016). According to the model, a subset of features from environmental stimuli and past events associated with present thoughts are temporarily activated within LTM. This embedded subset of information then enjoys a heightened state of activation while remaining vulnerable to time-based decay and similarity-based interference. A subset of the activated features can be made further salient and integrated into coherent objects and scenes when placed under the focus of attention, which allows a deeper semantic analysis of stimuli. The focus of attention is said to be limited to somewhere between three and five representational units (Cowan, Chen, & Rouder, 2004), which may be single-featured items or “chunked” items with multiple features (e.g., shape, color, location, orientation; Cowan, 2005).

The EP model assumes a limited-capacity domain-general central attentional controller (Cowan, 1999). Its role is to supervise covert processes that serve to maintain information over time by reactivating decaying memory representations via subvocal rehearsal, as well as × activation × way of the focus of attention. These activation procedures have been found to have an observable cost to processing tasks within a dual-task paradigm, such as drop in accuracy on nonverbal choice reaction time (RT) tasks with increasing concurrent verbal memory load (Chen & Cowan, 2009).

Temporary information in working memory is therefore represented within this hierarchical system. LTM representations are initially activated by incoming stimuli and information is then further activated within the focus of attention where it must be maintained. Once information leaves the focus of attention it begins to decay, and this decay can only be combated by reactivation within the focus of attention or through subvocal rehearsal. Although items represented within activated LTM memory are partially protected from decay, interference between items can occur based on overlapping features between individual items.

## Comparisons Between the Theoretical Views

In the present work, the three theoretical views we have described were compared in terms of the effects of processing on storage and vice versa, in a dual-task setting in which a verbal recall task is combined with processing in a different domain. A conundrum that must be appreciated to understand our approach is that all three of the views are capable of predicting interference between tasks under some circumstances. In the MCM approach, if the capacity of verbal storage is reached, additional items can be saved by recoding the information in visuospatial terms (or semantic representations), at the expense of visuospatial or semantic aspects of processing. In the TBRS approach, any attention needed for processing conflicts with attention needed for refreshing of the items to be retained. Finally, in the EP approach, the limited capacity of the focus of attention must be shared between items to be remembered and the goals, procedures, and data for processing. Given this convergence between approaches, a comparison of the models depends on more specific predictions and suppositions related to the experimental tasks.

The detailed predictions from the three theoretical frameworks will be presented after the task methods. Crucially, these methods incorporate key features that were intended to avoid some procedural differences across labs that might have given rise to contrasting results between testing sites. One aspect of working memory that is widely accepted is that its capacity varies from one individual to another, even if there are debates about how that individual variability should be measured. However, in many studies in which working memory load is manipulated, the task demands in different conditions are the same for all participants. This means that for someone with a high working memory capacity, an experimental manipulation intended to impose a high cognitive load, might, for them, actually be a low load relative to their capacity. Conversely, for someone with a low working memory capacity, what is deemed to be a low cognitive load in an experiment might, for this individual, effectively be a high cognitive load. By averaging the results across participants, in one lab that happens to recruit high capacity individuals, they might observe little or no effect of increasing the load of a single-task, or of requiring a processing task to be performed while retaining a memory load. In labs that happen to recruit lower capacity individuals, there will be very clear effects observed for cognitive load and of dual-task manipulations. We addressed this possible sampling error in two ways. One was to run each experiment in parallel in two independent labs that have previously reported contrasting results, and to use identical equipment and software to rule out subtle, but potentially important differences between labs. More importantly, in all experiments we measured the memory span and processing span for each participant. Then the memory load without and with a processing task was set at the span-level for each participant. Likewise, the processing load without and with a memory load was set at the level of the processing span for each participant. This process of adjusting, or titrating, cognitive demand according to the span of each participant is commonly used by labs that work within the MCM framework (e.g., Doherty & Logie, 2016), but tends not to be adopted by other labs.

**Figure 1 fig1:**
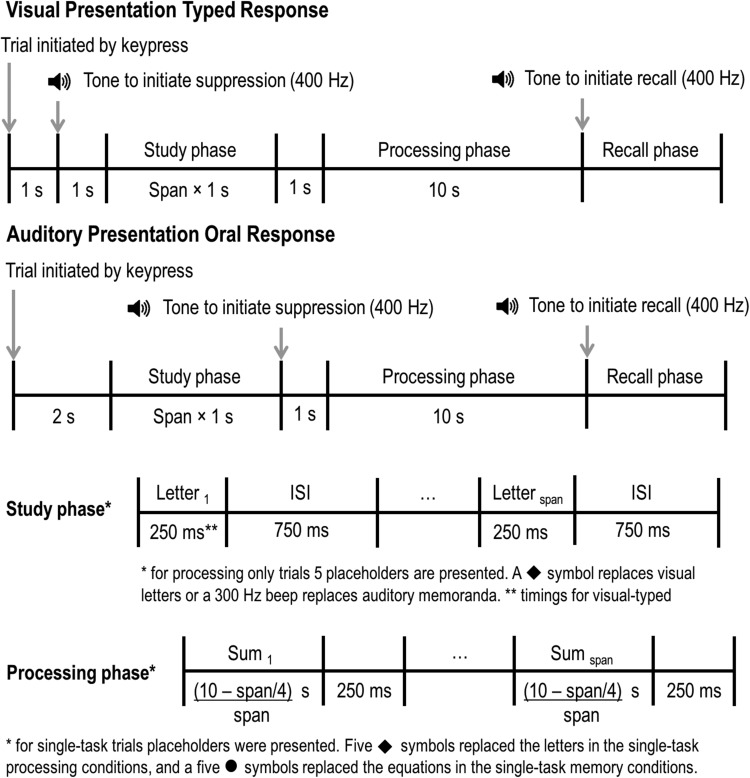
General trial sequences for Experiments 1–4, for visual/typed and auditory/oral presentation and recall conditions. The “tone to initiate suppression” only occurred in the articulatory suppression conditions. ISI = interstimulus interval.

A second important procedural detail is the extent to which trade-offs between memory and processing arise because of input and output conflicts when the two tasks are performed concurrently, or incompatibility between input modalities or output modalities, rather than because they require overlapping cognitive resources. Two tasks might mutually interfere because they both involve visual input, or both require an oral or keypress response. So, presenting verbal material visually and requiring an oral response, or presenting verbal material aurally and requiring a written response will require more cognitive operations than if the input and output modalities are more compatible, that is, aural input and oral response or visual input and written/typed response. We can avoid input and output conflicts by using a memory preload, with the processing task performed during the retention interval. Again, the extent to which these procedural details are considered varies across laboratories. Therefore in our experiments we avoid these potential artifacts by contrasting conditions in which there is aural presentation and oral recall of verbal memoranda with visual presentation and typed recall of these memoranda, without and with a visually presented processing task with a speeded single keypress response during a retention interval. This is illustrated in Figure 1.[Fig-anchor fig1]

Finally, when comparing single- and dual-task conditions, in some experiments, the single-task conditions always come first, or the order of single- and dual-tasks is counterbalanced across participants. The former approach could lead to practice effects on the tasks that could reduce the potential impact of requiring dual-task performance. The latter approach could lead to half of the participants showing a dual-task trade-off, because of unfamiliarity with each task and with performing two tasks together when the dual-task condition comes first, and the other half showing no such trade-off. We avoided these potential problems by requiring single-task performance before and after the dual-task condition. Comparing before and after single-task allowed an assessment of whether practice effects were evident in the tasks being combined. Also, the procedure for assessing span on each task acted to familiarize participants with each task before assessing single- and dual-task performance, and this should help to reduce the impact of task practice. In all of the experiments reported here we observed either null or small practice effects between the first and second single-task blocks, but crucially these practice effects did not change the observed patterns of statistically significant dual-task effects. For this reason the results of these analyses of practice effects are reported in the online supplementary materials.

## Overview of Experiments

In the current article, we present the results of four experiments with young adults. These experiments were designed to address differences among the assumptions and associated predictions from the three theoretical frameworks regarding whether or how the combination of processing and remembering affects performance of each relative to when they are each performed on their own. The theories also predict different effects of AS on visually or aurally presented verbal memory stimuli due to differences in the number of components or subsystems each framework contains.

In all of the experiments reported here, the focus was on how processing during a memory retention interval affects, or is affected by, serial ordered recall of a verbal memory preload when both the memory load and the processing load are set at the measured span (titrated) for each individual. The memory task involved presentation of a random letter sequence, followed by a blank retention interval (single-task) or a processing task (dual-task), then serial ordered recall of the letter sequence. The processing task involved speeded verification of simple arithmetic. The materials for each task were chosen to be compatible with testing English-speaking (U.K.) participants and French-speaking (Swiss) participants. The tasks were performed without or with AS, for reasons given later in the predictions from each theoretical framework. In line with our earlier discussion about possible procedural artifacts, in Experiments 1 and 3, the memory list was presented visually and recall responses were typed on the computer keyboard. In Experiments 2 and 4, the memory list was presented aurally and participants recalled the list orally. In Experiments 1 and 2, titration of span was carried out without AS, while it was carried out under AS in Experiments 3 and 4. For each experiment we tested differential predictions from each of the three theoretical frameworks.

## Experiment 1

The starkest contrast between the theories is MCM’s assumption that, with healthy adults, storage and processing can occur in parallel with little to no effect on performance in either task (e.g., Logie & Duff, 2007), particularly if tasks are titrated according to each participant’s individual abilities (e.g., Doherty & Logie, 2016; Logie et al., 2004), while both TBRS (e.g., Barrouillet & Camos, 2010; Barrouillet et al., 2004, 2007) and EP (e.g., Chen & Cowan, 2009; Cowan & Morey, 2007) argue for interference effects due to a shared central resource. MCM also argues for a visual store to support memory for visually presented verbal material (see Logie, 1995; Logie et al., 2000, 2016; Saito et al., 2008) and use of mnemonics (e.g., Logie et al., 1996; Paivio & Csapo, 1969) that can have a small effect on concurrent processing accuracy when rehearsal is prevented by AS, and so predicts more complex interaction effects than the additive main effects predicted by TBRS, and different patterns of interactions than the slot-based capacity of temporary memory argued by the EP theory. Experiment 1 aimed to investigate different predictions from each theory for the effects on a visually presented verbal memory task and a visually presented verbal processing task of performing both memory and processing together relative to performing each on its own, and also the effect of AS on the presence or magnitude of these effects.

### Method

This experiment, and all subsequent experiments, were approved by the ethics committees for The University of Edinburgh, The University of Fribourg, and The University of Geneva. The general trial sequences for all experiments are shown in Figure 1.

#### Participants

Participants were recruited from the student populations at the University of Edinburgh, United Kingdom, and the Universities of Fribourg and Geneva, Switzerland. They received different honoraria in each country due to concerns about differing motivation for cash rewards in each location. In the United Kingdom, participants were compensated for their time with an honorarium of £12. In Switzerland, participants were either offered cinema vouchers (equivalent to 16 CHF) or course credit. Sixty-four participants were recruited in total, 32 from each country (48 female and 16 male, *M*_age_ = 22.19, *SD* = 2.56). The sample size in each lab was selected to be comparable with previous research in the working memory literature, but to consist of a relatively large sample when compared to previous MCM, TBRS, and EP research.

#### Apparatus

Because the experiment was conducted across laboratories, efforts were made to ensure that the same equipment was used in each location. Each lab was equipped with the same model of laptop running PsychoPy (Version 1.84.2; Peirce, 2007), connected to the same models of external monitor, headphones, and button boxes. Due to differences in British English and Swiss French keyboard layouts, different models of keyboards were used at each site. PsychoPy settings and external monitors were set so that text stimuli were presented with an approximate vertical visual angle height of 1.3°. The same equipment and settings were used for all other experiments described in this article. The experimenter remained in the room during the experiment.

#### Procedure

The session began with a recognition task, in which participants were shown letters on screen and immediately typed the presented letter. Data from the pretest served as a check that the memory stimuli were sufficiently distinguishable from each other, and are reported in the online supplementary materials. The pretest was followed by the memory and processing titration conditions, which set the load levels for the single- and dual-task conditions for each participant. Participants completed the single- and dual-task conditions without and with AS, with half the participants completing the no-AS condition first and half starting with the AS condition. In each no-AS and AS block, participants started a single-task memory block and a single-task processing block consisting of 10 trials each (the order of the memory and processing blocks were also counterbalanced). This was followed by two blocks of 10 dual-task trials, followed again by two single-task blocks of memory and processing. Each participant therefore completed 40 single-task memory trials (20 without and 20 with AS), 40 single-task processing trials (20 without and 20 with AS), and 40 dual-task trials (20 without and 20 with AS).

##### Memory and processing titration procedure

Before the experimental conditions, both memory and processing loads were titrated to each participant’s individual abilities. The titration conditions followed a “staircase” procedure, in which the demand of a task was increased or decreased depending on a participant’s performance. Sixteen trials were presented in total, in pairs of two set at each level of demand, starting at five items for both tasks. If accuracy across a pair of trials was ≥80%, the demand of the task was increased for the next two trials; if accuracy was below 80% the demand was decreased. If a participant passed the final two trials (i.e., the eighth pair, Trials 15 and 16), and these two trials were the highest “level” they had reached up until that point, then additional pairs of trials were administered until failure to reach the 80% correct criterion. Participants’ memory and processing spans were recorded as the highest level at which they achieved 80% accuracy or above. Three practice trials were given at the start of each titration, with demand set to four items. Memory and processing titration were completed without AS in this experiment.

##### Single-task memory

The same set of letters was used for both English and French stimulus sets, which contained all the letters of the alphabet except vowels (to reduce pronounceability of memory sequences), and multisyllable letters from either language (*w*, *y*). The letter *z* was also excluded due to the desire to maintain parity with the stimulus sets for WoMAAC aging studies conducted across U.K. and U.S. laboratories, as *z* is pronounced differently in British and American English. Lists were randomly generated for each trial, without replacement. Participants initiated each trial with a button press, which was followed by a 2-s interval. Letters were then presented in the center of the screen sequentially for 250 ms each, with a 750-ms interstimulus interval (ISI). Therefore, the study phase lasted *n* × 1,000 ms. The onset of the last letter was followed by a two second interval, followed by a 10-s retention interval that consisted of five circles flashed on the monitor at a rate of one every 2 s, with a 250-ms ISI. Following the retention interval a 400-Hz tone sounded to prompt recall. Participant recalled items using the keyboard, and were able to pass on a letter by pressing the *0* key.

The AS conditions proceeded in much the same way, except that 1 s before the presentation of the first letter, a 400-Hz tone sounded to prompt participants to begin repeating “ba” at a rate of two per second (see Figure 1). Before each AS condition participants were presented with a tone playing twice every second to demonstrate the speed they should be repeating “ba.” Participants were instructed to cease AS when they heard the second tone (after the 10-s interval), and recall the memory items by typing them on the keyboard. To be clear, AS commenced prior to the start of the presentation of the memory sequence, and continued until after the filled or unfilled retention interval. This procedure was important for the MCM, which assumes that AS disrupts the use of phonological encoding and subvocal rehearsal of the visually presented letter sequence.

##### Single-task processing (arithmetic verification)

The processing task required participants to verify simple equations (e.g., “3 + 5 = 8, correct/incorrect?”). These equations were randomly generated for each trial, with each equation having a 50% probability of being presented with a correct solution. Participants initiated trials with a button press, after which they heard five 250-ms-long, 300-Hz “placeholder” beeps played once every second. Two seconds after the onset of the final beep, the first equation appeared for (10,000/*n*) – 250 ms (where *n* is the number of items to be presented), followed by a 250-ms ISI, then the next equation. Following the presentation of the final equation, a 400-Hz tone played to signify the end of the trial. Participants pressed a button marked with a “tick” (or “check”) for correct equations, and a button marked with a “cross” for incorrect equations (as they appeared on the screen). The task progressed whether the participant responded within the presentation time or ISI or not, that is, the sums remained on screen during their entire presentation window, and the ISI always occurred in full, regardless of the RT of the participant.

In the AS condition, a 400-Hz beep preceded the first 300-Hz placeholder beep to prompt participants to begin repeating “ba–ba–ba.” They were instructed to cease AS once they heard the second 400-Hz beep.

##### Dual-task

The single-task memory and processing procedures were designed to match the timing of the dual-task condition with the use of placeholder beeps or circles. Dual-task trials therefore proceeded in a similar fashion to the single-task memory condition, both without and with AS, except that instead of the placeholder circles appearing during the 10-s retention interval the arithmetic verification task appeared. Participants were instructed to complete both tasks, with no importance being placed on one task or the other by the instructions or by the experimenter. Participants were given three practice trials before the first 10 experimental dual-task trials were presented. The demand for the dual-task practice trials was set at one item below each participant’s span.

#### Predictions

Although each of the theoretical frameworks incorporates different assumptions, and therefore makes different predictions, none is a formal computational model and therefore the predictions are qualitative. The predictions refer to whether or not an effect is expected to be present, and whether any such effect will be small, medium, or large. Because the models cannot make specific predictions for the size of effects, particular emphasis was placed on predicting the size of effects in relation to other factors within the experiment (e.g., the size of the dual-task effect compared to the AS effect), and in later experiments predicting effect sizes in relation to previous experiments. The hierarchical models we describe in the upcoming analysis section estimate a random participant effect standard deviation, therefore summarizing the average difference between participants in the dependent variable (i.e., accuracy, or more specifically the log odds of a correct response). It is therefore possible to specify the size of effects arising from experimental designs by placing them on a scale of differences due to individual differences. WoMAAC partners were asked to generate their predictions with this scale in mind.

Predictions were specified in terms of small, medium, and large effects. Translating these into a common scale we used conventional criteria to refer to effects on the scale of expected individual differences (Cohen, 1988). Consequently, 0.2 of the average difference between individuals represents a small effect, 0.5 a medium effect, and 0.8 a large effect. These values were chosen as reasonable for effect sizes in research on memory (Morris & Fritz, 2013).^1^ To supplement the description of each account’s predictions simulated data conforming to the described expectations were generated and plotted and can be found on the OSF. Although each framework was required to generate predictions on the full set of variables, some predictions were speculative and not central to a particular theory. For example, the TBRS model has in the past largely focused on costs on memory, so predicted effects of dual-tasking on processing were generated from what the model would ideally expect when attention is split between tasks. Predictions were also generated in each theory’s proponents own chosen format: MCM and TBRS predictions focused on previous findings in the working memory literature, while EP generated predictions based on a simple capacity model created specifically for this experimental paradigm. The mathematical model generated by EP is available to view on the OSF, while a written summary of it is reported here for easy comparison with the predictions from the other theories. Table 1 summarizes the predictions made by each of the theories, and the full descriptions of these predictions are described in the next sections.[Table-anchor tbl1]

#### Multiple components

In the MCM, serial-ordered recall with visual presentation of a letter sequence is assumed to reflect (a) translation of the visually presented items into a phonological code; (b) the involvement of the phonological loop, comprising a passive phonological store and subvocal articulatory rehearsal to retain both item and serial order information as phonological codes; (c) visual encoding of the letters in a visual cache or temporary visual memory that can support item and order information; and (d) activation of representations of the visual and phonological information (of item, but not order) about the letters in LTM. All elements are thought to contribute to the observed span score. However, phonological encoding will dominate span performance when subvocal articulatory rehearsal is available. For memory above the span levels that are typical of healthy adults, there is thought to be an additional contribution from a range of mnemonic strategies such as chunking or semantic associations.

Visually presented items for arithmetic verification are assumed to involve activation of arithmetic knowledge in LTM and a decision process together with initiation of a manual response. None of these aspects of the task is thought to require use of the phonological loop, and so no effect of AS on processing is predicted by the MCM.

Visually presented memory items may be disrupted by the arithmetic verification task during the retention interval due to the concurrent activation in LTM of arithmetic knowledge and of letter representations. In addition to these disruptive effects, there may be an additional small disruption to memory because of the visually presented arithmetic disrupting the contents of the visual cache. The overall disruption will be seen as a small effect size because the operation of the phonological store and articulatory rehearsal will be unaffected by visually presented arithmetic verification. This prediction is derived from previous studies that have shown no, or small dual-task costs when combining an at-span verbal memory preload with a processing task (e.g., Cocchini, Logie, Della Sala, MacPherson, & Baddeley, 2002; Logie et al., 2004), and evidence showing low correlations between processing and memory performance (e.g., Daneman & Hannon, 2007; Logie & Duff, 2007; Waters & Caplan, 1996).

MCM assumes that AS during the encoding and retention phases will prevent phonological encoding and articulatory rehearsal of the memory items, and encourage the use of visual codes (e.g., Logie et al., 2000, 2016; Saito et al., 2008). Memory for visually presented letters will be impaired, because of a lack of phonological encoding and articulatory rehearsal, but will only be a medium effect size and will remain well above floor through a combination of passive storage within the visual cache, and activation of letter representations in LTM.

For dual-task with AS, memory for visually presented items will be impaired with a medium effect size because of the use of visual codes to support memory even when there is a lack of phonological encoding and articulatory rehearsal. This means there will be a Dual-Task × AS interaction, with a larger dual-task effect under AS. The support from visual codes may be less effective than for memory alone plus suppression because of interference from the visual presentation and manual response for arithmetic verification.

Under AS, there will be a small dual-task effect on verification because of participants attempting to use mnemonic strategies for retaining the letters to try to compensate for the lack of articulatory rehearsal. Therefore, for processing, a small interaction is also predicted such that there is a dual-task effect only under AS.

#### Time-based resource sharing

Verbal memory span reflects the involvement of both the phonological loop and the executive loop in the TBRS model (see Camos et al., 2017 for a review). At span (single-task, no AS), participants should recruit all the resources at their disposal, that is, because the phonological loop is limited to about four letters, the executive loop is used to “boost” performance beyond this limit. Thus, performing a processing task that involves attention (i.e., addition verification task) should disrupt the maintenance of verbal information through the executive loop and lead to a poorer memory performance than in the single-task condition.

The addition of concurrent articulation will impair the use of the phonological loop, resulting in poorer recall performance. Previous experiments showed that such a reduction is stronger than the reduction produced by a concurrent attentional-demanding task (e.g., Camos et al., 2009). Thus, TBRS predicts a medium main effect of task and a large main effect of suppression. Finally, the joint impairment of the phonological and executive loops by a concurrent articulation and the addition verification task, respectively, should lead to additive effects, and to a minimum recall performance. This should constitute a residual memory performance that remains when working memory maintenance mechanisms are blocked.

For the processing task, performing the addition verification task involves the executive loop. Because maintaining letters at span also involves the executive loop, a medium detrimental effect on processing should be observed in the dual-task condition compared with the single-task condition. AS should not have any effect on addition verification, except if AS induces a small attentional capture. In such a case, the addition of AS should result in a small reduction in processing performance. Therefore, two additive main effects are predicted, with the possibility of a small interaction to the extent that the addition task requires phonological processes.

#### Embedded processes

The EP model assumes that task relevant information from LTM is held in a heightened state of activation subject to decay and interference from other items with similar features. A subset of that activated information can be held in the focus of attention, which helps to overcome decay and interference. Additionally, a way to prolong and improve the maintenance of some verbal information with very little contribution of attention is through subvocal rehearsal.

To coordinate a verbal memory and verbal processing, dual-task participants must share the capacity of the focus of attention between these tasks. Compared to single-task performance, dual-task accuracy on memory and processing is predicted to be lower due to the need to split attention between these two tasks. Both tasks are assumed to benefit from subvocal rehearsal, and so an effect of AS on both tasks is predicted. However, memory performance also benefits from both rehearsal during encoding (as there is no AS during encoding for visually presented memory items) and visual sensory memory (due to memory items being presented visually). While rehearsal prevents time-based decay, visual sensory memory supports performance by providing additional storage while also freeing up the focus of attention for storage of other memory items. Likewise, the arithmetic task is assumed to rely on some mechanisms that are not relevant to the memory task (likely well-learned mathematical rules that can be recalled from LTM). This task also benefits from visual sensory memory, as the use of this separate storage frees up the focus of attention for processing.

These different factors contributing to single- and dual-task performance for each task lead to a set of predictions based on the overlap in shared mechanisms for each tasks. To make these predictions, some assumptions need to be made regarding the behavior of participants: (a) that participants are motivated to use all available resources to complete tasks and (b) that the attentional costs of the processing task can be expressed in terms of the number of items held in the focus of attention, as it is with the memory task. Although the theory does not specify the allocation of attention between tasks, when encouraged to make a guess at the allocation, the protagonists of this theory simply guessed that participants would split attention and other shared resources equally between the memory and processing tasks.

In sum, based on the assumptions made by the model as to the separate and shared mechanisms utilized for the memory and processing tasks, EP predicts large dual-task and AS costs to both memory and processing tasks. The model also predicts a smaller dual-task cost under AS (i.e., a medium interaction effect), as the shared subvocal resource is no longer split between the two tasks in single- and dual-task conditions, so the dual-task costs are reduced compared to the no AS condition.

### Results

#### Analysis method

To avoid the potential pitfalls of conventional methods (e.g., ANOVA and other normal models can lead to spurious results, particularly in the interpretation of interaction effects Dixon, 2008; Jaeger, 2008), data were analyzed using generalized linear mixed effects models (Bolker et al., 2009). This method allowed modeling of non-normal response variables (via a logit link function) while also acknowledging that observations are nested within individuals (i.e., repeated measures). The analyses were conducted using the lme4 package (Version 1.1–17, Bates, Mächler, Bolker, & Walker, 2015), and the full analysis scripts for the experiments reported in this article are available on the OSF. List of memory items and sequences of sums were analyzed on a by item basis: that is, if a participant remembered/responded correctly to three out of four items in a list/sequence, then the log odds would be modeled on this performance. Although participants were able to answer pass for the memory task, these responses were simply coded as incorrect for the purposes of analysis.

As detailed in the previous section, WoMAAC partners provided effect size predictions, but the first step of our analyses involved reducing the complexity of models to effects of interest. Initially full models, with all main effects and interactions plus a random intercept for each participant, were fitted to the memory and processing data. For both memory and processing data the main effects were task (single vs. dual), AS (without vs. with), and site (Switzerland vs. United Kingdom), and all interactions, including the three-way Task × AS × Site interaction, were included. The first model comparison involved removing the highest order interaction (the three-way interaction), and comparing it with the reduced candidate model. Model comparison was based on Bayesian information criterion (BIC) values (Schwarz, 1978): If these values were lower for the candidate model, this was evidence for the removal of the effect and to use the new simpler model for future comparisons. Two-way interactions, and then main effects, were then considered in turn. Each two-way interaction and main effect was considered separately with a model containing all other effects (apart from already removed higher order effects). If model comparison favored the inclusion of an interaction, lower order interactions or main effects contained within that interaction were not considered for removal later in the chain. Summaries of the best-fitting statistical models from each experiment are reported in this article, but the full analysis script showing each step is available on the OSF.

#### Analyses

**Figure 2 fig2:**
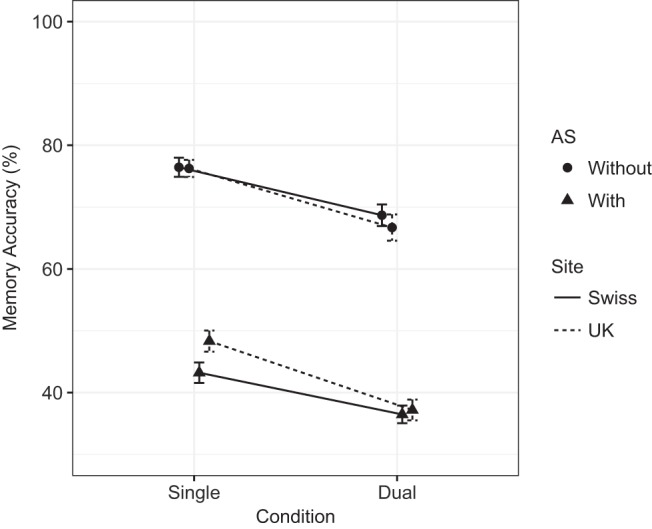
Mean memory accuracy with standard errors, across single- and dual-task conditions both with and without articulatory suppression (AS) in Experiment 1. Data are split by site (Swiss = Switzerland, UK = United Kingdom) to show interactions.

**Figure 3 fig3:**
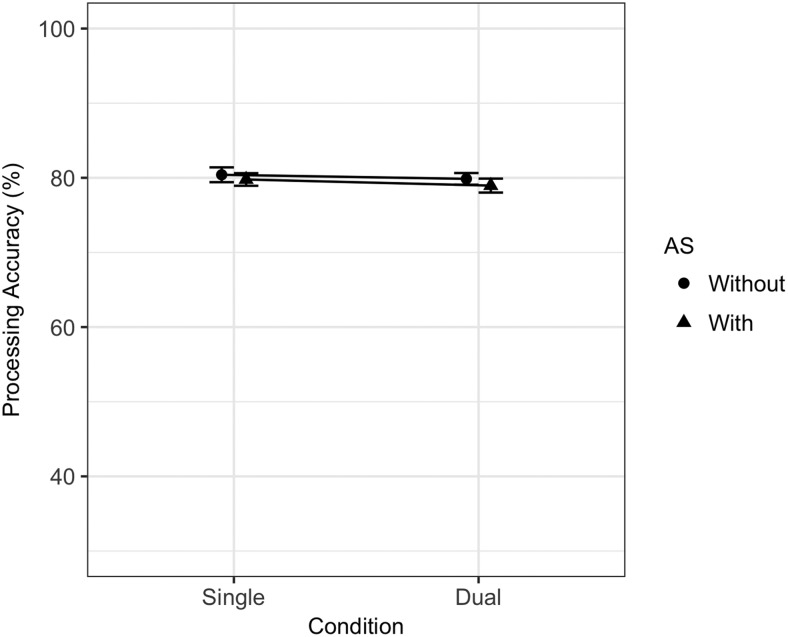
Mean processing accuracy with standard errors, across single- and dual-task conditions both with and without articulatory suppression in Experiment 1.

Mean memory span was 6.34 (*SD* = 1.28), and mean processing span was 8.00 (*SD* = 2.0). The best fitting memory and processing statistical models are summarized in Table 2. Because model comparison was conducted via BIC comparison, it is possible to calculate a Bayes factor comparing the winning statistical models to the next best candidate model. The Bayes factor in favor of the best fitting statistical model for memory was 31.34 (BIC for best fitting statistical model = 21,696.57; BIC for next best candidate model = 21,703.46), and for processing the Bayes factor in favor of the best fitting statistical model was 6,734.51 (BIC = 16,022.03; BIC for next best candidate model = 16,039.67). For memory, there were statistically significant main effects of dual-task (*ES*_scaled_ = −0.73) and of AS (−2.96). Although the effect of site was not statistically significant, the model comparison method described earlier resulted in the retention of Condition × Site and AS × Site interactions, both of which were statistically significant in the model (*ES*_scaled_ = −0.30 and 0.39, respectively). These interactions reflect a larger dual-task effect at the U.K. lab, and a smaller AS effect in the U.K. lab compared to the Swiss lab (note that the former interaction effect runs counter to the pattern that would be expected due to testing site bias). Figure 2 summarizes dual-task and AS effects split across labs and clearly demonstrates the source of the interactions is the larger single-task AS effect in the Swiss lab reducing the dual-task effect in the same lab. Contrary to the memory task, processing performance was not affected by either dual-task or AS manipulations (see Figure 3 for plotted data).[Table-anchor tbl2][Fig-anchor fig2][Fig-anchor fig3]

### Discussion: Summary of Experiment 1

All three theories made clear predictions for the outcome of Experiment 1, ranging from null effects (MCM), to additive effects of dual-task and AS (TBRS), to interactions between these two effects (MCM/EP). While each of the models predicted some of the observed effects, no account predicted the complete pattern of results.

A large dual-task effect was observed for memory performance. This does not fit with the predictions from the MCM of a small disruptive effect of processing on memory accuracy. Both TBRS and EP predicted the dual-task effect, yet both models predicted medium effect sizes where a very large effect size was observed. All three models predicted an effect of AS, though both MCM and EP predicted a medium effect size where a large effect as predicted by TBRS was in fact observed.

It is important to note that constrained effect sizes were used for predictions of small, medium, and large effect sizes (0.2, 0.5, and 0.8, respectively), so it may be considered more informative to compare each model’s predicted magnitude of dual-task and AS effects. Thus, TBRS correctly predicted that the dual-task effect would be smaller than the AS effect. MCM also predicted this pattern, but only because such a small effect of dual-task was predicted and the predicted size of the AS effect was still smaller than that observed. When forced to make a prediction of the relative effect sizes for dual-task and AS, EP assumed equal contribution of attention to rehearsal and processing and so predicted that these effects would be equal, which the data do not support.

MCM and EP both predicted Dual-Task × AS interactions with memory, though each predicted different patterns. Neither of these interactions was present in the data. Contrary to the MCM prediction, the effect of dual-task was present without and with AS, and the introduction of AS did not reduce the size of the dual-task effect as predicted by EP. That is, it appeared that the effects of dual-task and of AS were independent and additive.

TBRS predicted a medium dual-task effect and a small AS effect on processing with no interaction, while the EP model predicted the same Dual-Task × AS interaction as it did for memory where a smaller dual-task effect was observed under AS. Neither of these patterns was observed in the data. The MCM prediction of no dual-task effect on processing when there was no concurrent AS was accurate, yet the Dual-Task × AS interaction prediction was not confirmed, as AS did not introduce a statistically significant effect of task.

Finally, although large effects of AS and dual-task were found for memory performance, performance levels were still well above chance even when both dual-task and AS were required. This highlights a difference in emphasis between the three theoretical approaches, with MCM studies typically pointing to the size of the residual performance levels, even under high cognitive load, whereas TBRS and EP typically note the reduction in performance relative to baseline levels.

In summary, while each model predicted some trends no account provided a satisfactory approximation of all the observed data patterns. Where some models succeeded, for example TBRS and EP in predicting dual-task effects on memory, those same models failed to predict patterns in the processing task. The opposite pattern was partially true for MCM, where small dual-task effects on processing were predicted while the dual-task effects on memory were not. Considering that the models all specify some interplay between memory and processing in working memory, accurate or semiaccurate predictions of one half of the data are not sufficient to identify a “winning” framework.

## Experiment 2

Experiment 1 investigated the effect of the dual-task and AS on memory and processing, and found large effects of both on memory but no effects on processing. Experiment 1 featured visual presentation of memory items, which, according to the MCM, meant that these items were verbally recoded when there was no concurrent AS but that suppression prevented recoding leading to a dual-task effect. It occurred that there was a dual-task effect in both no-AS and AS conditions, but such a recoding hypothesis was only presented by the MCM and so may be of use when differentiating between the models. Experiment 2, therefore, replaced the visually presented memory task and typed recall with an aurally presented task and oral recall. In Experiment 2, we aimed to investigate whether the presentation format changed the pattern of statistically significant effects or increased/decreased the magnitude of these effects, as only the MCM would make strong predictions regarding differences in performance due to presentation format.

### Method

#### Participants

As mentioned previously, data collection for Experiments 1 and 2 ran concurrently, and so participants were recruited in the same way as described in Experiment 1, resulting in a sample of 64 participants, 32 from the United Kingdom and 32 from Switzerland (46 female and 18 male, *M*_age_ = 20.96, *SD* = 2.46). The samples for Experiments 1 and 2 were independent.

#### Procedure

The procedure for Experiment 2 proceeded in the same way as in Experiment 1, except for the substitution of an aurally presented task in place of the visually presented memory task, and participants responded orally rather than typing their responses.

#### Aurally presented verbal memory task

Memory task stimuli were generated using the built-in Apple OS × 10.11.4 voice. The American English voice “Allison” was used in the U.K. lab, and the French voice “Audrey” was used in the Swiss lab. The same list of letters from Experiment 1 was used in Experiment 2, and lists were again randomly generated for each trial without replacement. The auditorily presented memory task proceeded with the same timing as the visual presentation memory task in Experiment 1. Memory item onsets were separated by 1,000 ms, so that the study phase (as with Experiment 1) was *n* × 1,000 ms. Following the blank retention interval, or the retention interval filled with the processing phase, a 400-Hz tone prompted participants to orally recall the letters, saying, “pass” for any letter they could not remember. The experimenter typed the participants’ responses on a separate keyboard and monitor. Both the experimenter’s keyboard and monitor were out of view of participants.

In the AS conditions, the 400-Hz tone signaling the beginning of the AS component of the task was played 1,000 ms after the onset of the last memory item, rather than before the onset of the memory items as it had in Experiment 1. In Experiment 1 the AS during encoding was to maximize the use of nonphonological memory processes (i.e., to avoid phonological storage through recoding of the memory items); the encoding phase in the AS condition for Experiment 2 was presented in silence to maximize the likelihood of phonological storage of memory items—an important procedural consideration for the MCM.

#### Predictions

Data for Experiments 1 and 2 were collected concurrently, so the predictions for Experiment 2 do not take into account the findings from Experiment 1. The predictions for Experiment 2 are summarized in Table 1.

#### Multiple components

In the MCM, serial-ordered memory span with aural presentation of letters is assumed to reflect (a) a passive phonological store, (b) articulatory rehearsal, and (c) activation of representation of the letters in LTM for items, but not order. All three elements are thought to contribute to the observed span score. For memory above span levels that are typical for healthy adults, there is thought to be a contribution from a range of mnemonic strategies such as chunking or semantic associations.

When arithmetic verification is performed during a retention interval for an aural letter sequence, it is expected that the concurrent activation in LTM of arithmetic knowledge and of letter representations may result in some disruption of letter memory, because of a small contribution of LTM activation to item memory in auditory, serial order letter span. However, this disruption will not be statistically reliable because the operation of the phonological store and articulatory rehearsal will be unaffected by visually presented arithmetic verification. Thus, no dual-task cost is predicted. It is expected that there will be no effect on arithmetic verification of a memory preload of an at-span aurally presented letter sequence.

AS was added during a blank retention interval, but not during encoding. This is important because it allows for initial phonological encoding and rehearsal during presentation of the at-span letter sequence, but prevents articulatory rehearsal to retain the sequence during the retention interval. Memory for aurally presented letters will be impaired, showing a large effect of AS. Memory performance will remain above floor through a combination of passive storage within the phonological store and activation of letter representations in LTM.

When AS is added to visually presented arithmetic verification, it is anticipated that there will be no effect on verification performance. When AS is added to arithmetic verification after presentation (without suppression during encoding) of an aural preload of an at-span letter sequence, memory for the letter sequence will be impaired for the same reasons as for suppression during memory retention without arithmetic verification. The extent of the disruption will show as a large effect on memory. Thus there is no interaction predicted between suppression and task (single- vs. dual). There will be a small dual-task effect on verification under AS because of participants attempting to use mnemonic strategies for retaining the letters in an attempt to compensate for the lack of articulatory rehearsal. Therefore, for processing a small interaction is predicted such that performance should be below span (<80%) in the dual-task with AS condition.

#### Time-based resource sharing

The TBRS predictions for Experiment 2 are unchanged from Experiment 1, with medium effect of dual-task, a large effect of suppression on memory, and a small dual-task effect on processing.

#### Embedded processes

EP predictions for Experiment 2 closely match those from Experiment 1, and follow a similar set of assumptions. While in Experiment 1 letter memory was assumed to be supported by visual sensory memory, in this experiment memory performance is assumed to be supported by auditory sensory memory. Auditory sensory memory is assumed to be more efficient than visual sensory memory for verbal materials, providing an additional source of memory that does not have to be divided between storage and processing, and so medium dual-task and AS costs are predicted in contrast to the large effects predicted in Experiment 1. As in the previous experiment, EP predicts a medium interaction between dual-task and AS in which the dual-task cost under AS is smaller due to the fact that subvocal mechanisms are no longer utilized and therefore shared between memory and processing tasks.

### Results

Data from Experiment 2 were analyzed using the same methods as Experiment 1. Mean memory span was 6.52 (*SD* = 1.04), and mean processing span was 8.61 (*SD* = 2.00).

The best fitting statistical model for memory is summarized in Table 3, which displays coefficient estimates for each effect. The Bayes factor in support of this model over the more complicated candidate model (calculated using BIC values, winning model = 21,293.38, more complicated candidate = 21,309.80) was 3,677.54, and over 1 million for the simpler candidate model (BIC for simpler model = 22,739.29). There were statistically significant dual-task and AS effects. Scaling the dual-task effect in terms of average differences between participants, the effect of going from single- to dual-task results in an effect size of −1.21. The scaled AS effect size was −2.00.[Table-anchor tbl3]

**Figure 4 fig4:**
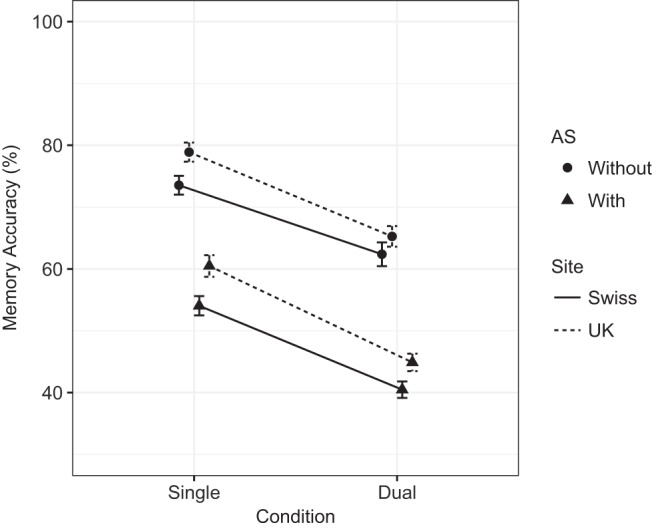
Mean memory accuracy with standard errors, across single- and dual-task conditions both with and without articulatory suppression in Experiment 2. Data are split by site (Swiss = Switzerland, UK = United Kingdom) to show the Dual-Task × Site interaction.

There was also a large effect of site (0.68), with U.K. participants performing better on the memory task than Swiss participants. As with Experiment 1, and contrary to what would be expected by site bias, there was also a slightly larger dual-task effect in U.K. participants (Condition × Site interaction, −0.34). Interpreting this main effect of site and interaction is straightforward when splitting participants’ performance across site (see Figure 4): The higher single-task performance in U.K. participants explains the larger dual-task effect. It is difficult to explain why Swiss participants did not perform at the 80% titration level, but because the interaction effect is small (and does not include the AS effect) it does not complicate interpretation of the overall data pattern.[Fig-anchor fig4]

**Figure 5 fig5:**
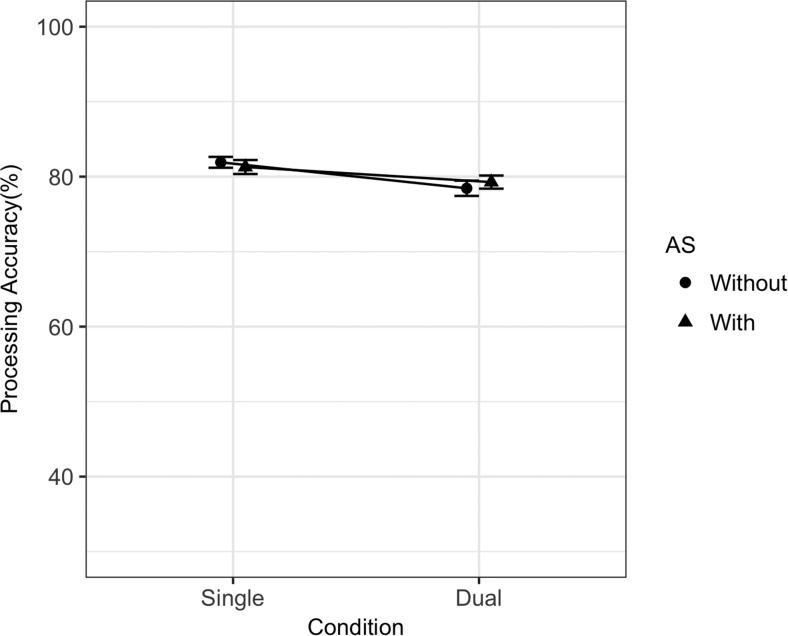
Mean processing accuracy with standard errors, across single- and dual-task conditions both with and without articulatory suppression in Experiment 2.

The best fitting statistical model for processing is also summarized in Table 3. Unlike memory performance, processing performance was only affected by the introduction of a dual-task (*ES*_scaled_ = −0.43). Note that this dual-task effect was not present in Experiment 1. Processing data are summarized in Figure 5. The Bayes factor in support of the best fitting statistical model was 4103.13 (BIC for best fitting model = 15,853.39, next best candidate model BIC = 15,870.03).[Fig-anchor fig5]

### Discussion

#### Comparison of Experiments 1 and 2

Memory and processing performance in Experiments 1 and 2 were compared using the same analysis method utilized for the separate analyses, except with the addition of a format between-subjects factor. The model comparison followed the same procedure of removing effects from the model and comparing BIC values, and the winning models for each task are summarized in Table 4. The Bayes factors supporting best fitting statistical models for memory and processing were 40.20 (BIC for winning model = 42,986.90, next best candidate model = 42,994.29) and 3,344.26 (winning model = 31,876.44, next candidate = 31,892.66), respectively.[Table-anchor tbl4]

For memory, aside from the clear effects of dual-task and AS (*ES*_scaled_ = −1.65 and −2.89), the best fitting statistical model also contained format interactions (though the main effect of format was not statistically significant). The Dual-Task × Format interaction reflects a larger dual-task effect for the auditory/oral task in Experiment 2 compared to the visual/typed task of Experiment 1 (*ES* = 0.57). However the AS effect was smaller for auditory/oral compared to visual/typed (*ES* = −1.38). There was also a Format × Site interaction as U.K. participants’ auditory/oral performance was higher than Swiss participants’ (this effect was also detected in the memory analysis of Experiment 1). For processing, there was an overall statistically significant dual-task effect (*ES* = −0.61) which was driven by the effect observed in the auditory/oral condition (Experiment 2) as evidenced by the Dual-Task × Format interaction (0.46).

#### Summary of Experiment 2

As with Experiment 1, a large dual-task effect on memory was observed with aural presentation of stimuli. MCM did not predict an effect of dual-task (either with or without AS), while TBRS and EP both predicted medium dual-task effects. The AS effect was predicted by all three theories, but only TBRS correctly predicted that this effect would be larger than the dual-task effect.

For processing, a medium dual-task effect was observed. TBRS predicted a small effect, and EP predicted a medium effect. MCM, however, predicted that the dual-task effect would only be present under AS (the same prediction as for Experiment 1), but this was not the case as no interaction between dual-task and AS was observed.

The between-experiment comparison revealed that the dual-task effect on memory was larger than that observed in Experiment 1. For processing, the between-experiments comparison confirmed the different patterns of data in Experiments 1 and 2 where a dual-task effect was only observed in the auditory/oral format condition. However, it is important to note the methodological differences between Experiments 1 and 2 relating to the onset of AS: For Experiment 1 (visual presentation), AS was carried out during the encoding phase, whereas in Experiment 2 the AS onset was after the presentation of the last memory item and before the processing phase/retention interval. This difference was important theoretically, as discussed in the introduction to Experiment 2. However, it may be that the differences in dual-task effect sizes were due to this difference in procedure, as AS may have interfered with encoding in Experiment 1 while having a start up cost that interfered with processing in Experiment 2.

MCM was the only model to propose different patterns of memory performance between Experiments 1 and 2, predicting a small dual-task effect with visual presentation and no effect for aural presentation. However, the opposite pattern was observed with a larger effect of dual-task on memory being observed in Experiment 2 compared to Experiment 1. While EP stated that different supporting memory processes were involved in visual and aural presentation tasks (i.e., visual and auditory sensory memory), the model did not predict that these differences would have an observable outcome on behavior. TBRS specifically predicted no difference between experiments, but differences were observed with a larger dual-task effect of memory in Experiment 2 than in Experiment 1, and a dual-task impact on processing in Experiment 2 that was not observed in Experiment 1. So, none of the three theoretical frameworks correctly predicted the full pattern of results observed across the two experiments.

#### Titration under AS

Experiments 1 and 2 revealed large dual-task effects on memory with both visual and auditory presentation formats, and null/small dual-task effects on processing. The three models had mixed success in predicting the patterns of results, though all three missed large trends in the data. Because Experiment 1 (visual/typed) most closely conformed to TBRS/EP for memory data, and to MCM for processing data, Experiment 3 adapted this procedure to investigate further the different assumptions regarding maintenance and processing and how maintenance and processing are affected by AS.

Each of the models makes some assumptions regarding the involvement of phonological/verbal rehearsal of memory items, and that these processes are affected by the addition of concurrent AS to the dual-task conditions. The goal of the titration procedure was to ensure that all participants were performing tasks set at appropriate levels of demand, but also to provide a reliable single-task measure of memory and processing performance. Titration of memory and processing tasks were completed without concurrent AS suppression, meaning that the memory task demand was adjusted to a level where memory was being supported by rehearsal.

Whereas all three models agreed that memory was supported by some form of subvocal rehearsal, only the MCM states that a small number of verbal memory items can be maintained with no requirement to rehearse or refresh (i.e., no attentional requirement). In MCM, subvocal rehearsal is said to “boost” memory performance beyond the capacity of this store. In Experiments 1 and 2 this means that, according to MCM, single-task memory performance is a product of not only attention-free storage but also rehearsal methods that are also affected by concurrent AS (Baddeley, Lewis, & Vallar, 1984; Murray, 1965). Experiments 3 and 4 aimed to test the MCM’s proposal of an attention-free verbal store by titrating memory under AS for both visual and auditory presentation formats in an attempt to more accurately measure the capacity of memory for verbal items when subvocal rehearsal is not available.

## Experiment 3

### Method

#### Participants

Participants were recruited in the same way as in previous experiments, half in the United Kingdom and half in Switzerland. The total sample consisted of 32 participants who had not taken part in either of the previous experiments (24 female and eight male, *M*_age_ = 21.72, *SD* = 2.25).

#### Procedure

The procedure for Experiment 3 closely resembled that of Experiment 1, with visual presentation and typed recall of memory items. The primary way in which the procedure deviated was that titration of memory and processing tasks was completed under AS. The trial procedures for memory and processing trials in the titration conditions followed the same timings as the AS conditions from Experiment 1. Single- and dual-task conditions were then completed in the same order as in previous experiments, however only data for performance under AS were collected.

#### Predictions

Predictions are summarized in Table 1.

#### Multiple components

The MCM predicted that there would be no subvocal rehearsal for the memory items because this would be prevented by the AS. There may be both phonological and visual encoding, with retention in passive, domain-specific temporary memory systems. Without suppression in previous experiments, rehearsal is assumed to be a strategy to boost temporary memory performance, and so span without suppression overestimates temporary memory capacity. Because rehearsal cannot be used under AS, the titrated spans will provide a more accurate measure of the capacity of the temporary memory systems. However, there might be attempts by some participants to use mnemonic strategies instead of rehearsal, and this would use a small amount of processing resource. Thus, MCM predicts that there will be at most a small dual-task effect, but possibly no effect on memory performance (contrary to Experiment 1), and no dual-task effect on processing performance (as was found in Experiment 1).

#### Time-based resource sharing

Under AS, memory span reflects the involvement of the executive loop in the TBRS model. Thus, performing a processing task that involves attention (i.e., the addition verification task) should disrupt the maintenance of verbal information through the executive loop and lead to poorer memory performance than in the single-task condition. The model therefore predicts a medium dual-task effect on memory.

For processing, performing the addition verification task involves the executive loop. Because maintaining letters at span also involves the executive loop, a detrimental effect on processing should be observed in the dual-task condition compared to the single-task condition. The TBRS model predicts a large dual-task effect on processing.

#### Embedded processes

In Experiments 1 and 2 participants were able to make use of subvocal rehearsal to reach a high span level during the titration procedure. The data from these previous experiments have led us to revise our account such that we no longer assume that rehearsal makes a contribution to processing. Thus, the manipulation of suppression and single- versus dual-task are assumed to be independent. Therefore, we predict a large effect of single- versus dual-task on memory in the present experiment where participants are titrated under suppression. Further, we predict that the dual-task cost on memory will be larger in this experiment relative to that found in Experiments 1 and 2. This is because we assume that the processing task consumes a constant “number of items” worth of attention and consequently it will have a greater cost in terms of proportion correct items recalled in position on the smaller list lengths obtained via titration under suppression.

For processing, there is a clear asymmetry in the data from Experiments 1 and 2. According to the EP account this is due to the preferential allocation of attention to the processing items as they appear at the expense of maintaining items in memory. Therefore, we predict no effect of single- versus dual-task on processing performance.

### Results

Data from Experiment 3 were analyzed using the same methods as previous experiments, yet because all the conditions were performed with suppression the process was simplified because there were only two main effects to consider: dual-task and site. Mean memory span under AS was 5.00 (*SD* = 1.00), and mean processing span under AS was 8.56 (*SD* = 2.00).

**Figure 6 fig6:**
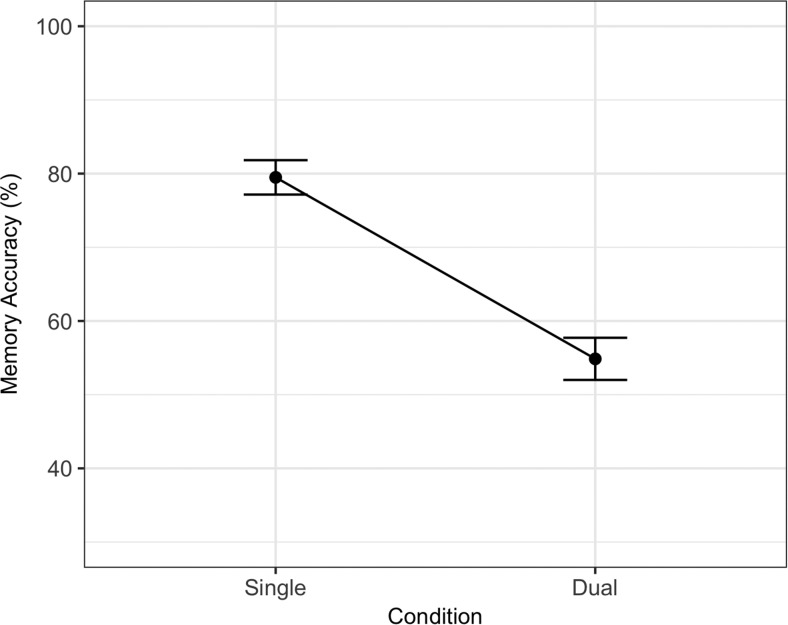
Mean memory accuracy with standard errors, across single- and dual-task conditions both with and without articulatory suppression in Experiment 3.

**Figure 7 fig7:**
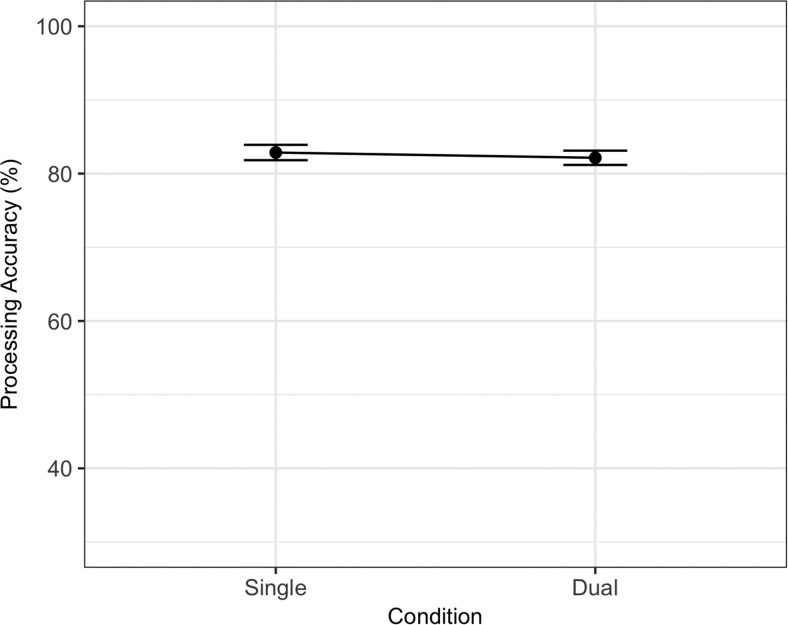
Mean processing accuracy with standard errors, across single- and dual-task conditions both with and without articulatory suppression in Experiment 3.

The best fitting statistical model for memory is summarized in Table 5 and contained a significant main effect of dual-task (*ES*_scaled_ = −1.64) and a Dual-Task × Site interaction (−0.49). The model comparison procedure produced a Bayes factor of 1.06 against the removal of the Dual-Task × Site interaction (BIC full model = 4,498.70, BIC for model without interaction = 4,498.81). As stated in the preregistered materials, we treated BIC as a binary choice in our model comparison procedure despite the inconclusive Bayes factor. The interaction reflects a larger dual-task cost in U.K. participants. There were no effects of dual-task or site on processing, with a Bayes factor of 361.41 supporting the removal of both of these factors (BIC for best fitting statistical model = 3,813.78, BIC for next best candidate model = 3,825.56). Memory and processing data are summarized in Figures 6 and 7.[Table-anchor tbl5][Fig-anchor fig6][Fig-anchor fig7]

### Discussion: Summary of Experiment 3

MCM predicted a small or null effect of dual-task on memory due to titration under AS resulting in a more accurate measure of the verbal memory store. Conversely, TBRS and EP predicted medium and large effects respectively. Contrary to MCM predictions, and in line with TBRS and EP, a large dual-task effect on memory was observed in Experiment 3.

Both EP and MCM predicted no effect of processing (as was observed in Experiment 1 with visual presentation and typed recall), though for different reasons. MCM predicted no effect due to separation of processing resources from memory, while EP predicted no effect on processing due to preferred allocation of attention to this more immediate task. TBRS predicted a dual-task effect on processing due to the involvement of the executive loop in maintaining memory items when subvocal rehearsal is prevented by AS. The results from Experiment 3 revealed no dual-task effect on processing—the same as was observed in Experiment 1.

## Experiment 4

Experiments 3 and 4 were run consecutively (unlike Experiments 1 and 2), and so some predictions for the latter experiment were influenced by the results from the former.

### Method

#### Participants

Thirty-two participants took part in Experiment 4, split evenly between the two labs as with previous experiments (23 female and nine male, *M*_age_ = 21.66, *SD* = 2.39). None of the participants had taken part in previous experiments.

#### Procedure

The procedure for Experiment 4 followed that of Experiment 3, with titration under suppression. However, Experiment 4 utilized the aural presentation and oral recall memory task from Experiment 2.

#### Predictions

Predictions are summarized in Table 1.

#### Multiple components

MCM assumes that AS will prevent rehearsal of memory items but will not prevent temporary phonological storage. Participants may attempt to use mnemonic strategies instead of rehearsal, which would use a small amount of processing resources leading to (at most) a small dual-task effect on memory and processing.

So, while a large dual-task effect on memory was observed for the visual/typed experiment with titration under AS (Experiment 3), a small or zero effect is predicted by MCM with auditory presentation because aurally presented memory items will have direct access to the phonological store. A small or zero dual-task effect is also predicted for processing, with any effect due to the aforementioned potential use of mnemonics.

#### Time-based resource sharing

The TBRS model predicts the same pattern of results as observed in Experiment 3. The TBRS model does not make specific predictions about differences in effect sizes, but states that titration with AS will result in participants relying to different degrees on the phonological and executive loops. The extent to which participants will rely on one mechanism or the other is not precisely predictable, but the switch from a visual/typed memory task to auditory/oral is not predicted to make a difference for the effect size, so TBRS predicts that the observed dual-task effect size for memory will be at least as large as the effect observed in Experiment 3 (−1.64). TBRS amends their processing task predictions to state only that a dual-task effect will be present (without specifying an effect size) because the theory does not specify working memory mechanisms or resources uniquely related to arithmetic verification, but that it induces an attentional cost that will disrupt refreshing via the executive loop.

#### Embedded processes

As with Experiment 1 and 3, EP again predicts that the dual-task cost will be *larger* in this experiment compared to that observed in Experiment 2, because processing task has greater cost in terms of the number of items in smaller lists.

The full analysis of Experiments 1 and 2 revealed a two-way interaction between format (auditory/oral, visual/typed) and task (single, dual). Given that this comparison was, in part, made between subjects, this interaction is not expected to replicate. Consequently, with regards to comparison to the follow-up study with visual presentation and typed response titrated under AS (Experiment 3), EP predicts that the dual-task cost for memory in this auditory/oral experiment will be at least as large if not larger.

For processing, EP predicts no effect of dual-task because of the preferential allocation of attention to the processing items in the retention interval. While Experiment 2 revealed a small dual-task cost for processing, EP does not predict a replication of this pattern in this follow-up experiment. A replication of a dual-task processing cost with auditory/oral presentation of memory items when we have not observed this with visual/typed (Experiments 1 and 3) would require further theoretical changes to the EP model.

### Results

**Figure 8 fig8:**
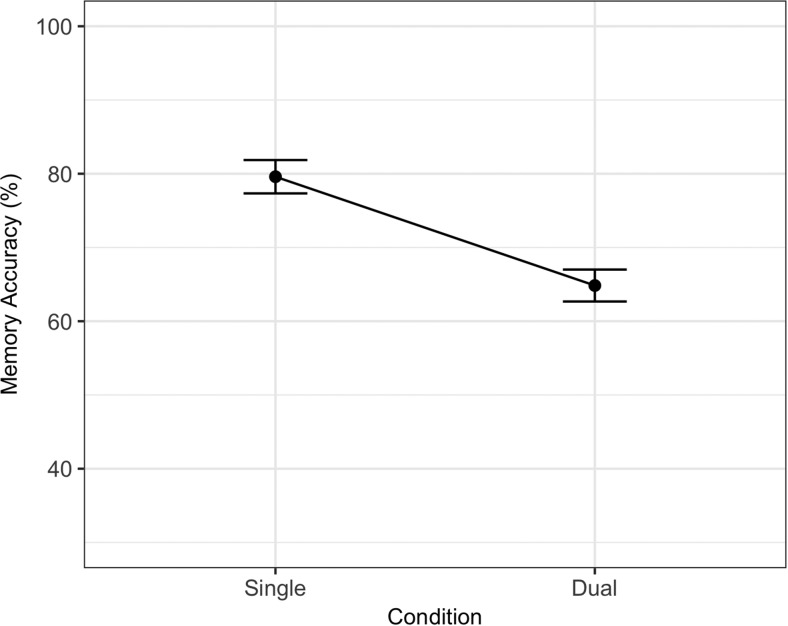
Mean memory accuracy with standard errors, across single- and dual-task conditions both with and without articulatory suppression in Experiment 4.

**Figure 9 fig9:**
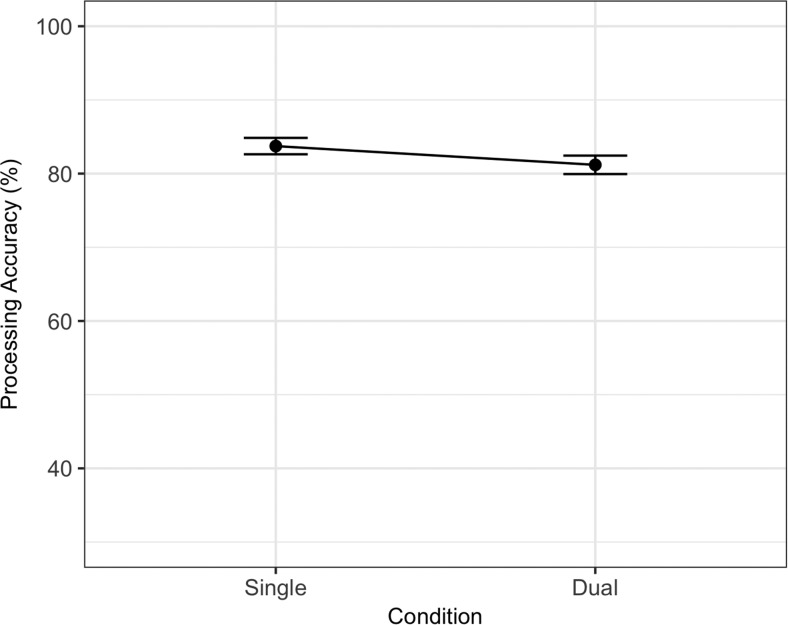
Mean processing accuracy with standard errors, across single- and dual-task conditions both with and without articulatory suppression in Experiment 4.

Mean memory span under AS was 5.20 (*SD* = 0.94), and mean processing span under AS was 7.66 (*SD* = 2.00). The best fitting statistical models for the memory and processing are summarized in Table 6, and data are summarized in Figures 8 and 9. Statistically significant dual-task effects were found for both memory (*ES*_scaled_ = −1.32) and processing (−0.42). For memory, a Bayes factor of 30.67 was found in support of the best fitting statistical model (BIC = 4,432.40) over the next best candidate model (BIC = 4,439.25). For processing the best fitting statistical model was supported by a Bayes factor of 33.78 (BIC = 3,648.41) over the next best candidate model (BIC = 3,655.45). As with previous experiments, no one theoretical framework correctly predicted the full pattern of results.[Table-anchor tbl6][Fig-anchor fig8][Fig-anchor fig9]

### Discussion

#### Full comparison of Experiments 1–4

Following completion of the fourth experiment, we found it pertinent to compare it with all previous experiments (and EP specifically made predictions regarding effect sizes between experiments). The analysis method followed the same procedure as for individual experiments, and the best fitting statistical models for memory and processing are summarized in Table 7. For memory, the Bayes factor in support of the full model was over a million (BIC = 56,563.51) compared to the next simplest candidate model (BIC = 56,614.56), and for processing the winning model was preferred by a Bayes factor of 106.17 (BIC = 39,313.58) over the next more complex candidate model (BIC = 39,322.91).[Table-anchor tbl7]

For memory, a number of statistically significant effects were found. The dual-task and format effects and the Dual-Task × Format interaction were observed in previous analyses. The titration effect and the Format × Titration reveal performance was *higher* with titration under AS. However, these effects are artifacts due to the differences in experimental designs of Experiments 1 and 2 versus 3 and 4: Mean performance was lower in the former two experiments because AS was added after titration levels were set. This means that in Experiments 1 and 2, on average, performance was lower as the mean was “pulled down” by the AS conditions. In Experiments 3 and 4, task demands were titrated under AS to 80% performance levels, and no additional load was added apart from dual-task.

Of interest is the Dual-Task × Titration Type interaction for memory, which reveals that the dual-task cost to memory was larger when titration was performed under AS (Experiments 3 and 4 vs. 1 and 2). Also, the three-way Dual-Task × Format × Titration Type interaction reveals a larger dual-task effect in Experiment 3 compared to other experiments.

#### Summary of Experiments 3 and 4

For both Experiments 3 and 4, MCM predicted a small or null effect of dual-task due to the memory task being titrated under AS, which was assumed to result in a more accurate measure of the verbal memory store by removing the “boost” to memory performance from rehearsal. However, a large effect of dual-task on memory was observed in both experiments (TBRS predicted a medium effect, while EP predicted a large effect). The between-experiment comparison revealed that this effect was in fact larger than the memory dual-task effects in Experiments 1 and 2, in which memory (and processing) were titrated without concurrent AS. This larger effect was predicted by the EP model, and was attributed to the fact that the attentional cost of the secondary task will result in a larger proportion of the shorter list lengths being forgotten (the shorter lists being a result of titrating under AS).

Experiments 3 and 4 also replicated the finding in Experiment 1 and 2, where a dual-task cost to processing was only observed when the memory stimuli were presented aurally. However, as discussed previously, it is difficult to ascertain whether this effect on processing is related to the presentation format of the memory task or due to the differences in AS onset. Specifically, the EP model predictions stated that this pattern might not be replicated in Experiments 3 and 4. MCM predicted no effect on processing in either Experiment 3 or 4, while TBRS predicted a large effect in Experiment 3 and a measurable effect (with an unspecified magnitude) in Experiment 4. As noted earlier, none of the theoretical frameworks predicted the pattern of observed results.

## General Discussion

Theories of working memory attempt to both explain existing behavioral data and to predict performance on tasks based on an assumed structure and functional organization of working memory. One of the starkest differences between working memory theories, and the focus of the present study, is the effects of dual-tasking on memory and processing performance; specifically whether or not retention of memoranda relies on continued or repeated access to an attentional resource, and the performance cost of this access to a concurrent processing task. The three theories investigated in this article provided predictions ranging from no effect of dual-task on memory or processing (MCM), to a linear trade-off between the two tasks (TBRS), and to an interactive pattern of effects due to the allocation of attention to different mechanisms supporting maintenance of memory items and verifying equations (EP). No one set of predictions matched the results obtained.

One of the possible explanations for differences between studies that found null/small dual-task effects in younger adults (e.g., Logie et al., 2004) and studies that found large trade-offs between processing and storage (see review Barrouillet & Camos, 2015) is that they could be due to a lack of titration in the latter body of research which instead focused on the maximum memory span achievable under dual-task rather than performance at span. For this reason, a titration procedure was utilized to ensure demand was set at appropriate levels for individual participants, therefore (according to the MCM) maximizing the likelihood that they would rely on specialized verbal stores rather than resorting to potentially attention-demanding strategies to cope with high task demand. The titration under suppression procedure in Experiments 3 and 4 aimed to further increase the use of a dedicated verbal store by removing participants’ ability to subvocally rehearse.

Despite setting memory and processing demand according to each participant’s individually measured spans, clear dual-task costs were observed in memory performance in all four experiments. This finding differed from previous MCM research with titrated demand that found little or no effect on memory (Cocchini et al., 2002; Doherty & Logie, 2016; Logie et al., 2004), and were more consistent with dual-task costs observed in previous EP and TBRS studies. In contrast, dual-task costs on processing were either not present or very small which was consistent with previous MCM studies on younger and older adults but not consistent with EP and TBRS.

Predictions from each framework were based on supporting evidence from the literature associated with each theoretical framework. The MCM predicted no dual-task effects based on previous findings (e.g., Doherty & Logie, 2016) and based on the assumption of a dedicated verbal store. As discussed previously, the assumption of a dedicated store dates back to the findings of Baddeley and Hitch (1974) in which dual-task costs were only observed at longer list lengths (hence the use of a titration procedure here to ensure list lengths, and processing task speed, were appropriate for individual participant’s abilities).

In Experiments 1 and 2 (for memory), only the prediction by MCM for the effect of AS for memory was supported by the data as a large effect of single- versus dual-task was observed in both experiments. TBRS predicted an additive effect of dual-task and AS on memory accuracy in Experiments 1 and 2, as was found. As summarized previously, the TBRS theory assumes that both storage and processing share, on a temporal basis, a common limited attentional resource through the alternating occupation of an executive loop while, for verbal maintenance, a domain-specific phonological loop can store some additional items to supplement the executive loop (Barrouillet & Camos, 2015). The predicted pattern of additive effects of dual-task and AS predicted by TBRS and borne out in the data from Experiments 1 and 2 is argued by TBRS to result from independent effects of diverting attention away from refreshing and preventing subvocal rehearsal. TBRS also predicted the relative magnitude of dual-task and AS effects, with AS having a greater impact on memory accuracy presumably due to greater reliance on subvocal rehearsal mechanisms when they are available, with the comparatively lower reliance on attention-based resources remaining great enough to evoke a substantial dual-task cost.

EP also correctly predicted dual-task (and AS) effects on memory in Experiments 1 and 2, yet attributed the cause to different mechanisms. The EP and TBRS approaches are consistent in many ways, most notably the use of attention to assist memory maintenance. It is therefore difficult to distinguish between the TBRS view in which the speed of attention-based refreshing explains capacity, and the EP view in which capacity may determine the speed of refreshing, with multiple items up to the capacity limit refreshed in parallel (for simulations of these models, see Lemaire, Pageot, Plancher, & Portrat, 2018).

EP also predicted an interaction between dual-task and AS, where a smaller dual-task cost under AS was expected. The fact that these interactions were not observed is relatively inconsequential for the framework as they were predicted based on arbitrary parameter values; there was no attempt to tweak the model or optimize it to get the best fit, as is often done in a model-fitting approach. Unlike TBRS, EP does not view the lack of interaction between dual-task and AS factors as evidence for separate systems, as it is not clear whether they would benefit performance in an additive or subadditive manner.

The MCM interpretation of the interim memory data from Experiments 1 and 2 was that allowing participants full use of subvocal rehearsal and some attention-demanding maintenance mechanism during the memory titration (i.e., titration being conducted in silence) resulted in spans representing input from additional resources (e.g., a visual store, mnemonics) rather than only the specialized short-term verbal memory store. This interpretation is supported by Doherty and Logie (2016) in which dual-task costs to processing were observed with no cost to memory spans, argued to be due to the fact that domain- or task-general attention-based sources could support memory performance (at a cost to the processing task) but that memory could not support processing due to the specialized nature of short term verbal storage resources. However, in Experiments 1 and 2 dual-task effects on processing were null and small respectively (Experiments 3 and 4 replicated the same pattern),^2^ suggesting no drop in performance to support memory. This contrast with the findings from Doherty and Logie (2016) merits exploration in future studies. It is notable that the lack of dual-task cost for processing is consistent with other previous MCM studies (Logie et al., 2004).

To further investigate the possible additional support from attention-demanding maintenance mechanisms, Experiments 3 and 4 aimed to reduce spans to be more representative of the capacity of the verbal store argued by the MCM. Titrating under AS, MCM presumed, would remove or reduce the ability of the participants to subvocally rehearse verbal memory items, and so performance would rely solely on the number of items they could store in verbal memory without rehearsal (auditory presentation), or on the support afforded by both a verbal and a visual store (visual presentation). For Experiments 3 and 4 (visual and auditory presentation respectively) MCM therefore predicted at most small effects of dual-task on verbal memory due to reliance on the verbal store and support from the visual store, with a small cost to memory performance potentially arising from the use of mnemonics being impaired by the processing task. However the MCM memory prediction was not supported by the data, as dual-task effects were larger than those observed in Experiments 1 and 2 were observed. The MCM interpretation of the observed effects speculates that, in the absence of rehearsal, people try to use mnemonic techniques to support performance, and this involves repeated access to LTM that is also required for the arithmetic verification task. It is notable that, in the original Baddeley and Hitch (1974) experiments, a memory load of 3 items resulted in no impact on a reasoning or language comprehension task performed during a retention interval. A memory load of six items did affect performance on the interpolated processing task, but only on response time, not on accuracy. It is possible that titrated span scoring generates an overestimate of the capacity of the phonological store, and as with the six-item memory list used by Baddeley and Hitch (1974), our titrated memory span exceeded that capacity.

Conversely, TBRS and EP both correctly predicted that the dual-task effects on memory in Experiments 3 and 4 would be larger than those observed in the previous experiments. According to TBRS, the larger dual-task effect on memory in Experiments 3 and 4 is interpreted as demonstrating the cost of diverting attention once tasks have been titrated to a level relying solely on this mechanism due to the prevention of subvocal rehearsal by AS. Forcing participants to rely on attentional refreshing results in span levels indicative of the lower capacity of this mechanism for maintenance of verbal memoranda compared to subvocal rehearsal. According to TBRS, the larger dual-task effect was observed in Experiments 3 and 4 because of greater reliance on refreshing throughout. Conversely, EP interpreted the larger dual-task effect to be due to the fact that the processing task costs memory a certain fixed number of items by taking attention, and that number of items results in a larger proportional loss when span has been reduced by eliminating the contribution of subvocal rehearsal. While both interpretations are similar the key difference is that TBRS specifies that the loss of memoranda during dual-task is due to participants reduced ability to attentionally refresh memoranda, while EP attributes forgetting to displacement of items from attention by the processing task.

The null/small dual-task effects on processing in Experiments 1–4 most closely match MCM predictions, as both TBRS and EP predicted medium/large effects. However, EP revised their predictions for Experiments 3 and 4, removing the assumption of an involvement of AS and interpreting the asymmetry in dual-task effects as being due to preferential allocation of attention to the processing task at the expense of memory performance. TBRS had assumed that because attention must be shared between memory and processing that participants would share “perfectly” between these two tasks and so the framework predicted the same dual-task cost would be observed in both. However, typical TBRS methodology has always placed a high priority on ensuring that participants are performing the processing task at a reliable level of accuracy (typically 80%) to ensure that the task reliably diverts attention away from refreshing memoranda. This emphasis typically leads to the removal of participants who perform below the accuracy criterion, though the majority of the sample is retained (e.g., Camos et al., 2009, between ∼ 1% and 5% of participants removed; Vergauwe, Barrouillet, & Camos, 2009, between ∼6% and 8%). It appears, therefore, that although TBRS predicted dual-task costs in both tasks, the asymmetry in which the dual-task costs are present only in memory is not inconsistent with previous TBRS findings in which there are often large dual-task effects on memory, yet the majority of participants are able to maintain processing performance >80% accuracy.

EP had predicted dual-task costs to processing based on other situations in which a processing task has, in fact, been affected by a memory task. For example, Chen and Cowan (2009) presented a three-choice task, in which participants had to press one of three buttons corresponding to a light on screen, with the task speed adjusted to produce errors. When this processing task occurred between digits to be recalled, the increasing memory load had a strong impact on three-choice performance. The results of Vergauwe et al. (2014), in which increasing memory loads affected processing task RTs, also influenced EP predictions on the speeded choice RT task used in this set of experiments. One difference between these findings is that the arithmetic verification task is more demanding (Vergauwe et al., 2014 featured relatively simple spatial and parity judgment tasks), and so EP speculates that it may not be possible for participants to divert attention during any one processing episode to engage in mnemonic restoration.

There was mixed success by each framework in predicting trends in the data, but all missed large trends in the data. Each theory requires some reconsideration of its core assumptions, or at least under what circumstances expected effects should be observed.

For example, MCM consistently predicted no dual-task effects on memory accuracy, and incorrectly predicted that the titration under suppression manipulation would remove the unexpected dual-task effect on memory observed in Experiments 1 and 2. MCM, however, was the only theory to predict small/null dual-task effects on processing, though the framework also predicted small Dual-Task × AS interactions that were not observed. These interactions were predicted as evidence for a trade-off from the processing resource to support memory when subvocal rehearsal was prevented/reduced by AS (small dual-task effects were tentatively predicted by the MCM in Experiments 3 and 4 for the same reason). Small yet statistically significant dual-task effects were only observed in auditory/oral experiments, in which the MCM would assume that aurally presented verbal memoranda had more immediate access to a phonological store and so performance would rely less on recruitment of additional resources or the use of mnemonics and so should predict smaller effects of dual-tasking on processing than when material is presented visually.

In sum, the MCM did not predict the large dual-task effects on memory accuracy, even when the experimental procedure was altered with the goal of maximizing the use of a dedicated verbal store. The MCM processing predictions were a close approximation of the processing data and the lack of small predicted interactions is not crucial for the framework which assumes separate resources for memory and processing. The between-experiment interactions cannot be easily explained by the framework or serve as clear cut evidence of the trade-offs in performance the theory assumes. By virtue of predicting small dual-task effects on memory, the MCM did expect the large residual performance in memory performance that was observed. MCM proposes that this residual memory performance is evidence for the involvement of multiple supportive mechanisms for memory, because if only subvocal rehearsal or attention supported verbal memory performance then the introduction of both these costs should have very substantially reduced performance to a larger absolute degree than observed. Although the effects on memory were medium or large relative to the intersubject variability, even the statistically large effects were small compared with the overall performance. For example, from Figure 2 (Experiment 1), the dual-task condition showed a ∼10% drop in mean proportion correct relative to single-task both with and without suppression. In Figure 4 (Experiment 2), the drop is around 15% in mean proportion correct. These drops in accuracy are comparable with previous dual-task studies in the MCM framework (e.g., Baddeley, 1986; Duff & Logie, 2001), although previous research analyzed data using ANOVA models, whereas here we analyzed data using more appropriate methods for accuracy data. While these effects may typically be labeled as “small” in terms of changes in proportion correct, predictions on proportion correct are only appropriate when dealing with computational models, and so scaling effects in the way described in this article provide information regarding the size of the dual-task cost in relation to a reliable metric, that is, between participant variability. To qualify predictions expressed in terms of proportion correct one solution might be for MCM to develop a computational model, or to adapt the existing qualitative model to predict effects scaled to between-participants variability.

Although the MCM expected large residual performance, it should be noted that neither TBRS and EP accounts predicted a performance drop to zero; TBRS would require both AS and a cognitive load of 1, that is, complete attentional capture, to predict floor performance. In fact, the residual memory performance observed in these experiments closely resembles that observed under extreme conditions of cognitive load (e.g., Barrouillet et al., 2004). Likewise, EP posits that participants are able to split attention between tasks while also benefiting from activations in LTM, and so would not expect floor performance with the dual-task procedure utilized in the reported experiments. While neither EP nor TBRS makes predictions about the size of the residual performance, even if they have implicit assumptions that allow a plausible explanation for the residual that was observed, MCM is more explicit in predicting a large residual. This illustrates a difference in emphasis between the theoretical frameworks, with the former two focusing on the dual-task costs, while the latter focuses on the substantial residual memory performance relative to modest dual-task effect costs to proportion correct. Also, the MCM assumption of separate storage and processing stores was based on previous findings where low correlations between memory and processing spans were observed (e.g., Daneman & Hannon, 2007; Logie & Duff, 2007; Waters & Caplan, 1996), and a post hoc analysis of the data from the current experiments reveals no statistically significant correlations between memory and processing spans (for Experiments 1, 2, 3, and 4, Pearson’s *r* coefficients were .24, .23, .2, and .01, respectively, all *p* > .05). The low level of shared variance between memory and processing spans, to the MCM, indicates evidence for separate components contributing to performance on each task and could explain the large residual performance observed in even the most demanding experimental conditions reported here. Again, the MCM focus on what performance remains and how separate working memory components could account for this performance further demonstrates differences in approaches between the theoretical frameworks and warrants further investigation.

The TBRS model successfully predicted both the presence of dual-task effects on memory, their relative magnitude to AS effects, and that the dual-task effect size would increase when span was measured under suppression. TBRS failed to predict the small/null dual-task effects, and the lack of AS effects, on processing. It remains unclear whether this theoretical framework requires modification to accommodate these findings. As already discussed, the asymmetric dual-task costs between memory and processing is not inconsistent with previous TBRS research. However, the lack of an effect is somewhat inconsistent with the findings of Vergauwe et al. (2014), where memory load was observed to affect processing RTs. Because processing titration relied on increasing the speed of the arithmetic verification task until participants’ accuracy dropped below 80%, it is logical to assume that any RT cost to processing performance should be reflected in accuracy. A post hoc analysis of RT revealed a small dual-task cost (see online supplementary materials to this article). This RT cost was either too small to impact speeded-response accuracy, or participants may be engaging in some speed/accuracy trade-off that preserves performance on the task enough to prevent a measurable drop in accuracy.

According to the TBRS model, a possible explanation for the lack of dual-task effects on processing (one that does not require the separation of memory and processing resources, or speculation of some representation-based interference based on Presentation/Recall × Processing Dual-Task interactions^3^), is that participants prioritized the addition verification task over the memory task. Studies on dual-tasking have established that interference between tasks can be modulated by priorities (Schumacher et al., 2001) and external cues play a role in the way participants select their goals (Altmann & Trafton, 2002; Jansen, van Egmond, & de Ridder, 2016). It is possible that the successive presentation of additions on screen and the requirement to produce immediate responses led participants to prioritize the verification task over the maintenance of letter lists. Vergauwe et al. (2014) detected dual-task effects on processing only after trials with imperfectly recalled lists were removed from the analysis: it may be the case that the effects resulting from resource sharing mainly appear when tasks are explicitly or implicitly given priority by participants (e.g., due to their immediacy) or by researchers (e.g., by designing paradigms that emphasize perfect or high performance on one or the other task within a dual-task paradigm). Accounting for prioritization phenomena within the TBRS model would require specifying the mechanisms by which attention is devoted either to maintenance or processing activities and what are the mechanisms that lead the executive loop to switch from one activity to the other, something that the current version of the TBRS model does not. For example, it might be imagined that remembering memory items is participants’ initial main goal in working memory tasks, and that the occurrence of a to-be-processed distractor on screen would trigger the reinstantiation of the task set associated with the concurrent task, thus leading attention to switch from maintenance to processing. Beyond this preliminary suggestion, what is needed is a temporally fine-grained description of the cognitive processes that successively take place during dual-task completion as well as the internal (volitional, strategic) and external cues that trigger them.

The EP framework (Cowan, 1988, 1999) has evolved since it was first proposed. Cowan (1988) left open the issue of how much semantic information is automatically analyzed and retained without attention, but the answer has to date appeared to be “little if any” (e.g., Conway, Cowan, & Bunting, 2001). Also, assumptions about attention and information storage have changed; for example, dual-modality memory task results of Saults and Cowan (2007) suggested that when participants cannot rehearse to-be-recalled items, memory is limited to three or four items. A psychometrically more thorough examination by Cowan, Saults, and Blume (2014) suggests that instead, participants first widen attention to take in three to four items in a set but then can quickly offload information to the activated portion of LTM. Cowan has long realized that the EP is a modeling framework to be filled in, not a complete model; an approach made clear by the revision of assumptions and predictions between Experiments 1 and 2 and Experiments 3 and 4 in this article.

Although the EP framework correctly predicted effects of processing on storage, and its magnification under AS, the aspect of the results most surprising for the framework is the absence of effects of concurrent storage on processing. A post hoc interpretation would concern the nature of the processing task, which might require attention but in a manner that is obligatory rather than optional. Previous studies suggest that simple arithmetic can involve direct retrieval from LTM as a preferred route of performance (e.g., Geary & Wiley, 1991), and other work suggests that this LTM retrieval is obligatory; people may not have the ability to modulate this use of attention to share with other tasks while the retrieval is ongoing (Craik, Govoni, Naveh-Benjamin, & Anderson, 1996; Schneider & Shiffrin, 1977). This assumption can be implemented without a change in the modeling framework but with an additional clarity in predictions, so that we would now predict that attention costs would accrue to processing as well as storage provided that the processing task was changed to one not requiring LTM retrieval (for a similar approach see Ricker, Cowan, & Morey, 2010). The outcome of such research examining different processing tasks in a dual-task design might not only explain the results reported here but may also inform future iterations of the EP framework, and/or help distinguish between MCM, TBRS, and EP accounts.

## Conclusion

The present work aimed to contrast predictions from MCM, TBRS, and EP theories of working memory by collaboratively designing a set of experiments for which (to the greatest extent possible) disparate predictions could be generated by each theory. We focused on the absence/presence/magnitude of dual-task effects on a pairing of verbal memory and verbal processing tasks, and on how AS modulated these effects. This research represents, to our knowledge, the first attempt at an adversarial collaboration to contrast working memory theories directly with the same experimental paradigm. Its main strength is the a priori design considerations made for each of the theories, resulting in outcomes that challenge the assumptions of all three models.

The experiments also highlight two novel challenges for adversarial collaborations. First, despite our initial assumptions based on the high level of debate in the working memory literature, it is difficult to design experimental procedures that result in clearly contrasting predictions from all three theories. The main difference between theories, at least for dual-task effects, is in how effects are interpreted. This is most evident in how EP and TBRS each explain the increased dual-task cost between Experiments 1 and 2 and Experiments 3 and 4. By challenging the three theoretical frameworks with the observed data patterns, the current experiments have highlighted the strengths and limitations of those frameworks, while providing new insights into how working memory functions under dual-task demands. However, to fully disentangle the subtle differences in interpretation will require future effort for new experimental designs. The differences between the theoretical frameworks are also highlighted by the tendency for MCM to focus on the substantial residual performance that remains even under very demanding dual-task conditions, whereas EP and TBRS focus on the presence of a drop in performance relative to single-task or low cognitive load demands, suggesting that the differences may not be as substantial as they appear. However, each of the three approaches would require modification to develop a more integrated account for the current set of data, for previous data sets generated within each framework and to generate more accurate predictions for future experiments.

Second, while the collaborative design process aimed to reduce post hoc interpretations of effects, such explanations are unavoidable. We do not, however, view this as a negative. Because the experiments were designed to take into consideration assumptions from each theoretical framework the scale of post hoc explanation is considerably reduced compared to what one might expect between competing theories researching and publishing work independently. Instead, the adversarial collaboration approach has resulted in a set of interpretations which rely on additional assumptions not directly tested here. These interpretations present a clear roadmap for future research; for example, whether task priority plays a role in the distribution of dual-task costs, if/how the input from additional resources supporting memory can be increased or reduced, and how the distribution of dual-task costs and/or the input from other mechanisms accounts for the residual performance in memory accuracy.

Our findings support statistically large dual-task costs to memory accuracy that favor a shared resource structure of working memory such as that proposed by TBRS and EP accounts, but with residual memory performance that may indicate input from other resources or mechanisms argued by the MCM. While this residual performance in and of itself is insufficient to distinguish a “winning” framework, both it and the asymmetry between memory and processing dual-task costs pose questions as to whether working memory can ever be explained by any one of these three frameworks, or whether some integrated combination of the three accounts will be needed to provide a comprehensive explanation of these and both previously published and future behavioral data.

## Supplementary Material

10.1037/xlm0000668.supp

## Figures and Tables

**Table 1 tbl1:** Summary of the Predictions From Each of the Three Models for Experiments 1–4

Effect	MCM	TBRS	EP	Observed
Experiment 1				
DT (mem.)	Small	Medium	Large	*ES*_scaled_ = −.73
DT (proc.)	Null	Medium	Large	*n.s*.
AS (mem.)	Medium	Large	Large	*ES*_scaled_ = −2.96
AS (proc.)	Null	Small	Large	*n.s*.
DT × AS (mem.)	Small	Null	Medium	*n.s.*
DT × AS (proc.)	Small	Null	Medium	*n.s.*
Experiment 2				
DT (mem.)	Null	Medium	Medium	*ES*_scaled_ = −1.21
DT (proc.)	Null	Medium	Medium	*ES*_scaled_ = −.43
AS (mem.)	Large	Large	Medium	*ES*_scaled_ = −2.00
AS (proc.)	Null	Small	Medium	*n.s*.
DT ×AS (mem.)	Null	Null	Medium	*n.s.*
DT × AS (proc.)	Small	Null	Medium	*n.s.*
Experiment 3				
DT (mem.)	Null/Small	Medium	Larger than Experiments 1 and 2	*ES*_scaled_ = −1.64
DT (proc.)	Null	Large	Null	*n.s.*
Experiment 4				
DT (mem.)	Null/Small	Equal to Experiment 3	Larger than Experiment 2	*ES*_scaled_ = −1.32
DT (proc.)	Null/Small	Effect predicted^a^	Null	*ES*_scaled_ = −.42
*Note.* MCM = multiple-component model; TBRS = time-based resource sharing; EP = embedded processes; DT = dual-task; mem. = memory; proc. = processing; AS = articulatory suppression; *ES*_scaled_ = scaled effect size; *n.s.* = not significant. Effect size labels were used to aid the generation of differential qualitative predictions. Summaries of the observed results are also listed.				
^a^ No specified effect size.				

**Table 2 tbl2:** Memory and Processing Analyses From Experiment 1, Displaying Coefficient Estimates and Standard Errors From the Winning Models for Each Task

Parameter	Task	
	Memory	Processing
Intercept	1.190*** (.091)	1.410*** (.048)
Dual-task	−.356*** (.034)	
AS	−1.436*** (.034)	
Site (Swiss/UK)	.010 (.129)	
Dual-Task × Site	−.143*** (.048)	
AS × Site	.191*** (.049)	
*Note.* AS = articulatory suppression.		
*** *p* < .01.		

**Table 3 tbl3:** Memory and Processing Analyses From Experiment 2, Displaying Coefficient Estimates and Standard Errors From the Winning Models for Each Task

Parameter	Task	
	Memory	Processing
Intercept	1.051*** (.083)	1.540*** (.054)
Dual-task	−.537*** (.033)	−.175*** (.024)
AS	−.890*** (.024)	
Site (Swiss/UK)	.304*** (.116)	
Dual-Task × Site	−.152*** (.047)	
*Note.* AS = articulatory suppression.		
*** *p* < .01.		

**Table 4 tbl4:** Mixed-Factorial Analyses Comparing Memory and Processing Performance Between Experiments 1 and 2, Displaying Coefficient Estimates and Standard Errors From the Winning Models for Each Task

Parameter	Task	
	Memory	Processing
Intercept	1.080*** (.087)	1.539*** (.052)
Dual-task	−.539*** (.029)	−.175*** (.024)
AS	−.941*** (.029)	
Format (AO/VT)	.086 (.122)	−.107 (.073)
Site (Swiss/UK)	.246** (.122)	
Dual-Task × Format	.185*** (.034)	.133*** (.035)
AS × Format	−.452*** (.034)	
Dual-Task × Site	−.147*** (.034)	
AS × Site	.104*** (.034)	
Format × Site	−.186 (.168)	
*Note.* AS = articulatory suppression; AO = auditory/oral; VT = visual/typed.		
** *p* < .05. *** *p* < .01.		

**Table 5 tbl5:** Memory and Processing Analyses From Experiment 3, Displaying Coefficient Estimates and Standard Errors From the Winning Models for Each Task

Parameter	Task	
	Memory	Processing
Intercept	1.422*** (.178)	1.582*** (.064)
Dual-task	−1.076*** (.087)	
Site (Swiss/UK)	.078 (.250)	
Dual-Task × Site	−.321*** (.119)	
*** *p* < .01.		

**Table 6 tbl6:** Memory and Processing Analyses From Experiment 4, Displaying Coefficient Estimates and Standard Errors From the Winning Models for Each Task

Parameter	Task	
	Memory	Processing
Intercept	1.428*** (.111)	1.696*** (.086)
Dual-task	−.759*** (.057)	−.182*** (.053)
*** *p* < .01.		

**Table 7 tbl7:** Mixed-Factorial Analyses Comparing Memory and Processing Performance in Experiments 1 Through 4, Displaying Coefficient Estimates and Standard Errors From the Winning Models for Each Task

Parameter	Task	
	Memory	Processing
Intercept	.725*** (.064)	1.536*** (.047)
Dual-task	−.585*** (.023)	−.176*** (.022)
Format (AO/VT)	−.251*** (.091)	−.097 (.061)
Titration (no AS/AS)	.696*** (.117)	.163*** (.063)
Dual-Task × Format	.201*** (.033)	.129*** (.031)
Dual-Task × Titration	−.172*** (.061)	
Format × Titration	.286* (.167)	
Dual-Task × Format × Titration	−.687*** (.088)	
*Note.* AO = auditory/oral; VT = visual/typed; AS = articulatory suppression.		
* *p* < .1. *** *p* < .01.		

## Footnotes

1 Upon analysis of the data, effects far larger than 0.8 were in fact observed. Because predictions of large effect sizes were based on this smaller value, the magnitude of predicted effects were unavoidably underestimated. However, because each framework made predictions based on this same scale, it was still possible to evaluate contrasting predictions when data were analyzed.

2 The differences in dual-task effects on processing across different memory presentation/recall formats were not predicted or easily explained by any of the three frameworks. A follow-up experiment had mixed success in replicating the pattern (i.e., dual-task effect on processing only in the auditory/oral memory condition), but this replication only occurred at the U.K. site. The effect remained small, and so we concluded that these small dual-task effects on processing are unreliable and possibly due to sampling effects. In any regard, these dual-task effects were always considerably smaller than for memory. The experiment is reported in the online supplementary materials to this article.

3 See the online supplementary materials to this article for the between-subjects follow-up investigation of these interactions.
