# Correction to Doherty et al. (2018)

**DOI:** 10.1037/xlm0000761

**Published:** 2019-09

In the article “Dual-Task Costs in Working Memory: An Adversarial Collaboration” by Jason M. Doherty, Clement Belletier, Stephen Rhodes, Agnieszka Jaroslawska, Pierre Barrouillet, Valerie Camos, Nelson Cowan, Moshe Naveh-Benjamin, and Robert H. Logie (*Journal of Experiment Psychology: Learning, Memory, and Cognition*, 2019, Vol. 45, Iss 9, pp. 1529–1551. http://dx.doi.org/10.1037/xlm0000668), the copyright attribution was incorrectly listed and should have published under the Creative Commons CC-BY license. The correct copyright is “© 2018 The Author(s).” All versions of this article have been corrected.

