# Supplementary material for: Dual-Task Costs in Working Memory: An Adversarial Collaboration
Source: J Exp Psychol Learn Mem Cogn. 2018 Nov 8;45(9):1529–51. doi: 10.1037/xlm0000668 (PMC6727883; doi:10.1037/xlm0000668)
Supplement: Supplementary file 1 [file DTcosts_AC_Supplementary.pdf]

Supplement to "Dual-task costs in working memory: An adversarial collaboration"

Jason M. Doherty, Clément Belletier, Stephen Rhodes, Agnieszka J. Jaroslawska, Pierre Barrouillet, Valérie Camos, Nelson Cowan, Moshe Naveh-Benjamin, and Robert H. Logie

Contents

Practice effects **2**

Pretest in Experiments 1 and 2 **4**

Between-subjects analysis of Visual/Typed and Auditory/Oral memory tasks **6**

Experiments 1-4 Reaction Time Analyses **7**

Practice effects

In order to investigate practice effects for memory and processing, accuracy in the dual-task conditions and the second single-task condition were compared to performance in the first single-task block. The winning statistical models from these analyses are summarised in Tables 1-4, and the full analysis script is available on the Open Science Framework (OSF). In brief summary, statistically significant practice effects were observed for memory only in Experiments 2 and 3. For processing, practice effects were observed only in Experiment 2. Crucially, where dual-task effects are reported in the main analyses, differences between first/second single-task blocks and dual-task conditions were closely matched. Meaning that dual-task costs cannot be accounted for by an increase in performance across the course of the experiment with high accuracy in the second single-task block resulting in the statistically significant dual-task effects when single-task blocks are collapsed together.

Table 1: Experiment 1 - Practice effects analyses for memory and processing.

	Task	
	Memory	Processing
Intercept	1.235*** (0.071)	1.410*** (0.048)
Dual-task (DT) vs. First single-task (ST1)	−0.481*** (0.044)	
Dual-task (DT) vs. First single-task (ST2)	−0.041 (0.053)	
AS	−1.601*** (0.050)	
DT/ST1:AS	0.289*** (0.060)	
DT/ST2:AS	0.449*** (0.070)	
Note:		*p<0.1; **p<0.05; ***p<0.01

Table 2: Experiment 2 - Practice effects analyses for memory and processing.

	Task	
	Memory	Processing
Intercept	1.235*** (0.071)	1.504*** (0.057)
Dual-task (DT) vs. First single-task (ST1)	−0.481*** (0.044)	−0.140*** (0.030)
Dual-task (DT) vs. First single-task (ST2)	−0.041 (0.053)	0.072** (0.035)
AS	−1.601*** (0.050)	
tempCondition2:AS1	0.289*** (0.060)	
tempCondition3:AS1	0.449*** (0.070)	
Note:		*p<0.1; **p<0.05; ***p<0.01

Table 3: Experiment 3 - Practice effects analyses for memory and processing.

	Task	
	Memory	Processing
Intercept	1.301*** (0.132)	1.582*** (0.064)
Dual-task (DT) vs. First single-task (ST1)	−1.083*** (0.072)	
Dual-task (DT) vs. First single-task (ST2)	0.354*** (0.091)	
Note:		*p<0.1; **p<0.05; ***p<0.01

Table 4: Experiment 4 - Practice effects analyses for memory and processing.

	Task	
	Memory	Processing
Intercept	1.432^{***} (0.119)	1.652^{***} (0.094)
Dual-task (DT) vs. First single-task (ST1)	-0.763^{***} (0.072)	-0.137^{**} (0.065)
Dual-task (DT) vs. First single-task (ST2)	-0.007 (0.086)	0.090 (0.077)
Note:		*p<0.1; **p<0.05; ***p<0.01

Pretest in Experiments 1 and 2

Experiments 1 and 2 featured discrimination tests for memory stimuli. Error frequencies are shown in Figures 1 and 2.

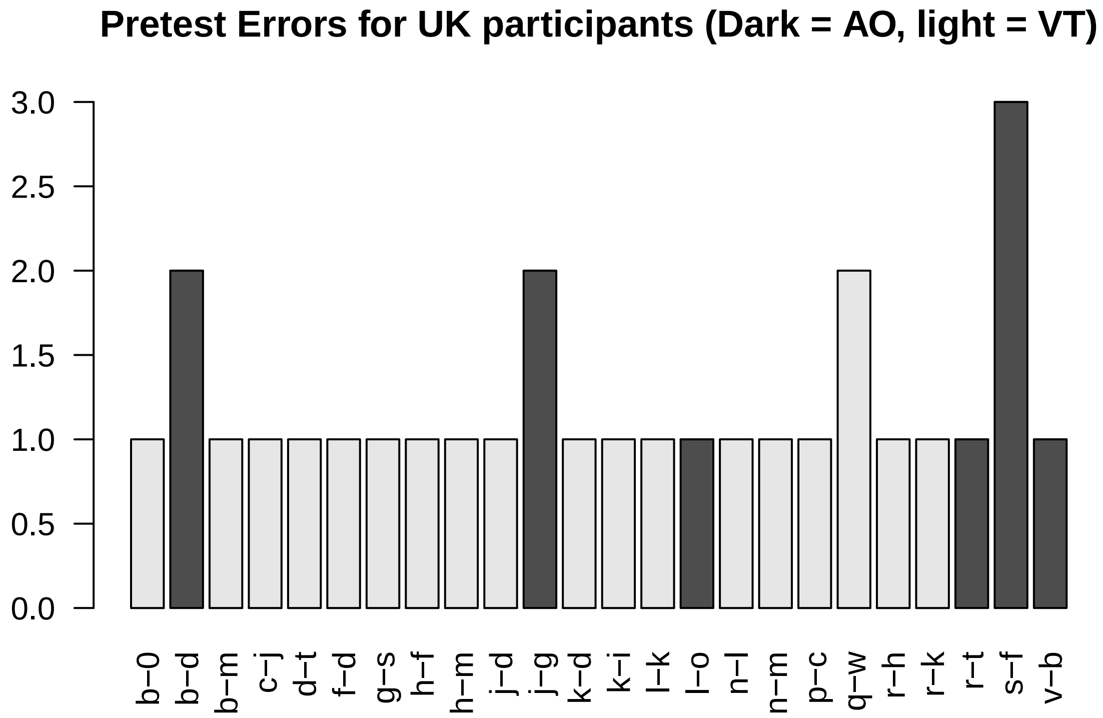

Figure 1: Pretest Errors for UK participants in Experiments 1 and 2.

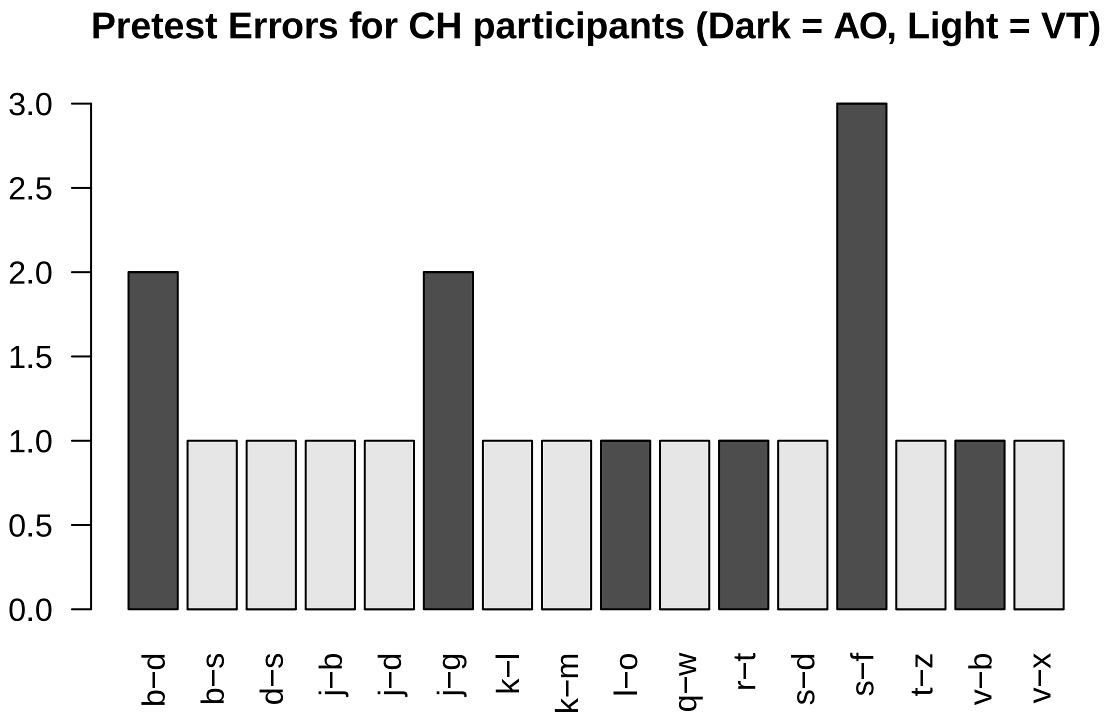

Figure 2: Pretest Errors for CH participants in Experiments 1 and 2.

Between-subjects analysis of Visual/Typed and Auditory/Oral memory tasks

To investigate the between-experiment interactions from Experiments 1 and 2, we conducted a follow up experiment directly comparing visual/typed (VT) and auditory/oral (AO) presentation/recall formats between subjects. The main motivation for this experiment was to directly contrast the different patterns of *processing* performance observed between Experiments 1 & 2 and between Experiments 3 & 4.

The experimental procedure for each format was the same as in Experiments 1 and 2, except that the articulatory suppression (AS) onset was the same for both formats. Sixty-four participants took part in the study, evenly split across sites and AO/VT conditions (49 female and 15 male, mean age = 21.13, SD = 1.93).

The results of the memory and processing analyses are summarised in Table 5. The crucial analysis was that of processing accuracy, where a dual-task cost was only observed in the UK participants, with no dual-task:format interaction. The small size of this dual-task effect, the small dual-task effect on processing observed in Experiments 1 and 3 from the paper, and the lack of interaction in the between-subjects analysis leads us to conclude that dual-task effects to processing are small and unreliable. It is probable that differences between these results and the findings of other experiments are due to factors such as sample effects, and/or differences in statistical power.

Table 5: Experiment 5 - Between subjects experiment contrasting visual/typed and auditory/oral memory presentation/recall formats.

	Task	
	Memory	Processing
Intercept	1.540^{***} (0.121)	1.384^{***} (0.109)
Dual-task vs. First single-task	-0.529^{***} (0.024)	-0.048 (0.038)
AS	-1.107^{***} (0.051)	
Format (AO vs. VT)	-0.166^{***} (0.054)	
Site (CH vs. UK)	-0.452^{***} (0.169)	0.093 (0.134)
AS:Format	0.164^{**} (0.070)	
AS:Site	0.275^{***} (0.069)	
Format:Site	0.503^{***} (0.177)	
AS:Format:Site	-0.349^{***} (0.097)	
conditionDT:siteUK		-0.175^{***} (0.051)
Note:		*p<0.1; **p<0.05; ***p<0.01

Experiments 1-4 Reaction Time Analyses

Processing task reaction times were analysed via Bayesian ANOVA. Reaction time (RT) data are summarised in Figures 3-6. All combinations of the main effects and interactions were tested, and the winning model and corresponding BF are shown in the figure captions.

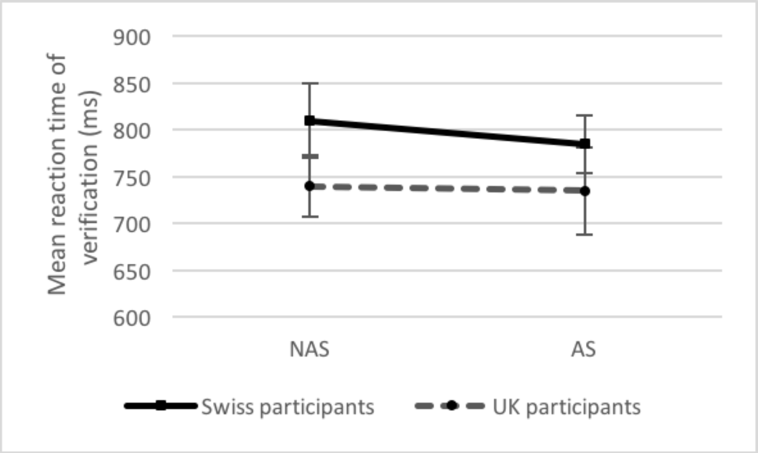

Figure 3: Experiment 1 - Mean RTs plotted by suppression (no AS, AS) and site, with 95% confidence intervals. The winning model contained an effect of AS and Site on RT, BF = 1.6e+9

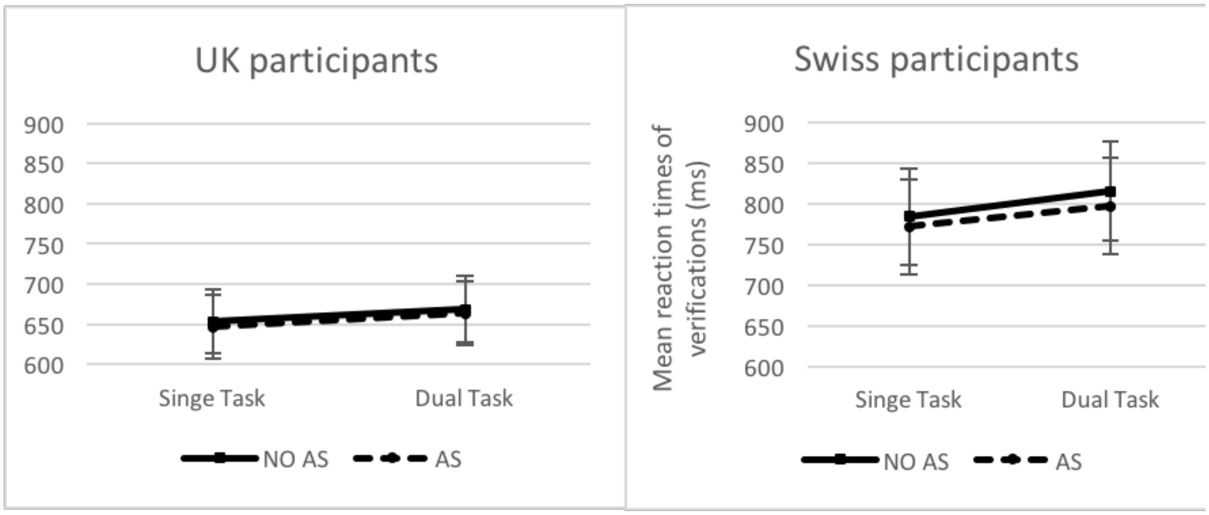

Figure 4: Experiment 2 - Mean RTs split by UK and CH participants. RTs are plotted by task and AS. The winning model contained effects of Site, Dual-task, and AS on RT, BF = 1.02e+21.

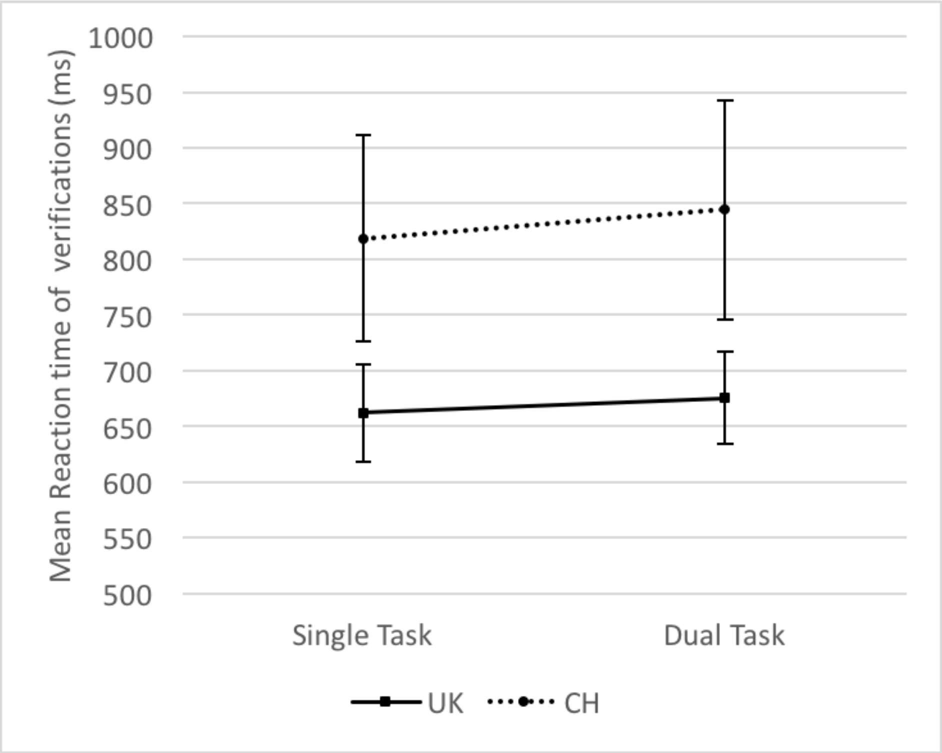

Figure 5: Experiment 3 - Mean RTs plotted by condition and site, with 95% confidence intervals. The winning model contained an effect of Dual-task and Site on RT, BF = 249.99e+10

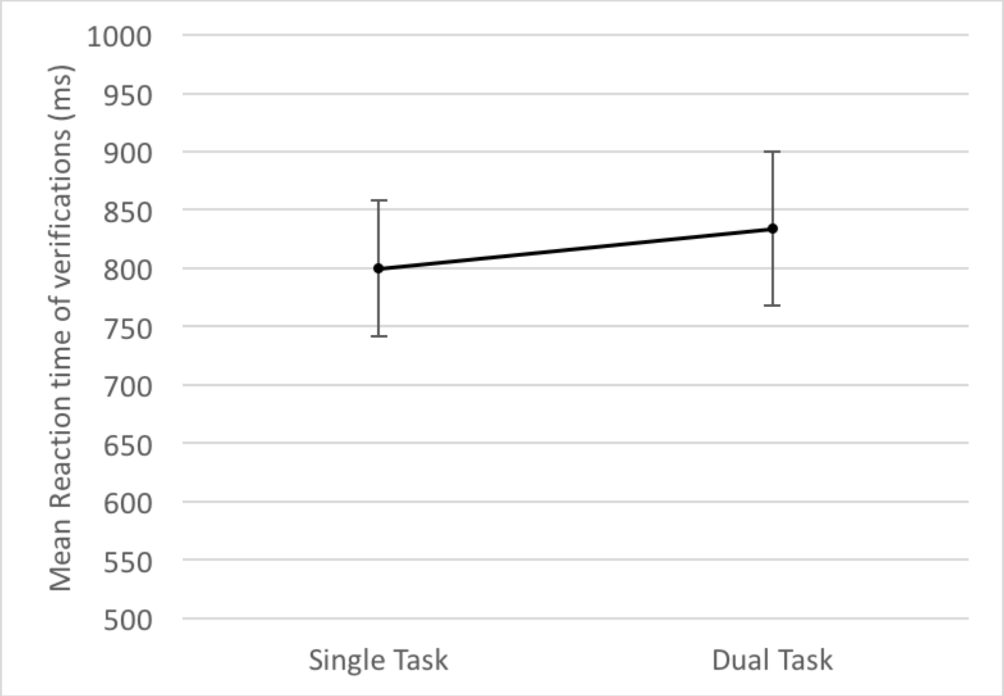

Figure 6: Experiment 4 - Mean RTs plotted by condition, with 95% confidence intervals. The winning model contained only an effect of Dual-task on RT, BF = 249.99e+10
